# Transcriptional profiling of pediatric cholestatic livers identifies three distinct macrophage populations

**DOI:** 10.1371/journal.pone.0244743

**Published:** 2021-01-07

**Authors:** Sarah A. Taylor, Shang-Yang Chen, Gaurav Gadhvi, Liang Feng, Kyle D. Gromer, Hiam Abdala-Valencia, Kiwon Nam, Salina T. Dominguez, Anna B. Montgomery, Paul A. Reyfman, Lorena Ostilla, Joshua B. Wechsler, Carla M. Cuda, Richard M. Green, Harris Perlman, Deborah R. Winter

**Affiliations:** 1 Division of Pediatric Gastroenterology, Hepatology, and Nutrition, Department of Pediatrics, Ann and Robert H Lurie Children’s Hospital of Chicago, Chicago, Illinois, United States of America; 2 Division of Rheumatology, Department of Medicine, Northwestern University, Chicago, Illinois, United States of America; 3 Division of Pulmonary and Critical Care Medicine, Northwestern University, Chicago, Illinois, United States of America; 4 Division of Gastroenterology and Hepatology, Northwestern University, Chicago, Illinois, United States of America; Texas A&M University, UNITED STATES

## Abstract

**Background & aims:**

Limited understanding of the role for specific macrophage subsets in the pathogenesis of cholestatic liver injury is a barrier to advancing medical therapy. Macrophages have previously been implicated in both the mal-adaptive and protective responses in obstructive cholestasis. Recently two macrophage subsets were identified in non-diseased human liver; however, no studies to date fully define the heterogeneous macrophage subsets during the pathogenesis of cholestasis. Here, we aim to further characterize the transcriptional profile of macrophages in pediatric cholestatic liver disease.

**Methods:**

We isolated live hepatic immune cells from patients with biliary atresia (BA), Alagille syndrome (ALGS), and non-cholestatic pediatric liver by fluorescence activated cell sorting. Through single-cell RNA sequencing analysis and immunofluorescence, we characterized cholestatic macrophages. We next compared the transcriptional profile of pediatric cholestatic and non-cholestatic macrophage populations to previously published data on normal adult hepatic macrophages.

**Results:**

We identified 3 distinct macrophage populations across cholestatic liver samples and annotated them as lipid-associated macrophages, monocyte-like macrophages, and adaptive macrophages based on their transcriptional profile. Immunofluorescence of liver tissue using markers for each subset confirmed their presence across BA (n = 6) and ALGS (n = 6) patients. Cholestatic macrophages demonstrated reduced expression of immune regulatory genes as compared to normal hepatic macrophages and were distinct from macrophage populations defined in either healthy adult or pediatric non-cholestatic liver.

**Conclusions:**

We are the first to perform single-cell RNA sequencing on human pediatric cholestatic liver and identified three macrophage subsets with distinct transcriptional signatures from healthy liver macrophages. Further analyses will identify similarities and differences in these macrophage sub-populations across etiologies of cholestatic liver disease. Taken together, these findings may allow for future development of targeted therapeutic strategies to reprogram macrophages to an immune regulatory phenotype and reduce cholestatic liver injury.

## Introduction

Macrophages are a heterogeneous and plastic cell population that respond to environmental signals in various cholestatic liver diseases [1–3]. Tissue-resident macrophages of the liver, also termed Kupffer cells, are self-renewing cells that are present in the liver at birth and promote tolerance in homeostasis [4]. In the setting of liver injury, tissue-resident macrophages can adopt a pro-inflammatory state and additional monocyte-derived macrophages may be recruited from the peripheral circulation to the liver [5–8]. This leads to a heterogeneous population of macrophages that may have distinct functions in disease.

Prior studies have presented conflicting evidence for a role of macrophages in obstructive cholestasis. Recruited monocytes have been shown to have a protective role against infection in the setting of murine bile duct ligation [9]. In contrast, C-C chemokine receptor type 2 (CCR2)-mediated recruitment of monocyte-derived macrophages in a murine model of primary sclerosing cholangitis has been implicated in the mechanism of liver injury and fibrosis [10]. Similarly, macrophages have been associated with the pathogenesis of murine parenteral nutrition-associated cholestasis via toll-like receptor 4 (TLR4)-mediated activation [11] and production of interleukin-1 beta (IL-1β) [12]. Furthermore, reduced farnesoid x receptor (FXR) signaling is thought to induce activation of the macrophage inflammasome in cholestasis and endotoxemia, thereby promoting IL-1β release and increasing immune susceptibility in cholestasis [13]. However, the precise subsets of macrophages responsible for cholestatic liver injury and repair have not been fully characterized.

Macrophages have also been more specifically implicated in biliary atresia (BA), an obstructive cholangiopathy of infants thought to arise from an aberrant immune response to a self-antigen. While there are two major forms of BA, isolated BA (iBA) and syndromic BA (BASM) with associated malformations, evidence supports a similar antigen-driven immune response in both subtypes [14]. Evidence supporting a role for macrophages in this mal-adaptive immune response include the observation that increased numbers of macrophages correlate with poor prognosis in human BA [15–18]. Hepatic macrophages are also increased in the rotavirus-induced murine model of BA [19]. In addition, macrophage depletion in a murine model of BA improved bile duct obstruction [20]. These studies demonstrate a central role for macrophages in promoting liver injury in BA but fail to identify the specific pathogenic versus pro-restorative macrophage subsets.

In our current work, we define human liver macrophage heterogeneity in pediatric cholestasis by analyzing single-cell RNA-sequencing (scRNA-seq) from patients with cholestasis from BA or Alagille Syndrome (ALGS, a non-immune etiology of obstructive cholestasis) and comparing these with non-cholestatic pediatric liver and previously published normal hepatic macrophages [21]. We identify novel hepatic macrophage subsets in obstructive cholestasis that are distinct from non-diseased macrophages by leveraging the ability of scRNA-seq to define cell sub-populations. We further demonstrate reduced expression of regulatory genes across all cholestatic macrophage subsets that may contribute to loss of immune tolerance in cholestasis. Taken together, our results lay the foundation for future mechanistic studies and development of macrophage-specific immune modulatory therapies.

## Experimental methods

### Human tissue samples

Formalin-fixed, paraffin-embedded liver tissue sections from non-diseased donor liver (n = 5), and BA (n = 6), and ALGS (n = 6) patients at the time of liver transplantation were obtained from the pathology archives of Ann & Robert H. Lurie Children’s Hospital of Chicago. Fresh liver tissue was obtained from the explanted liver of 3 patients with cholestatic liver disease (2 with BA and 1 with ALGS) and 1 patient with a hepatic tumor at the time of liver transplantation. Laboratory data was collected retrospectively from the hospital admission for liver transplantation. Written informed consent was obtained from each patient’s legal guardians including in the study. The study protocol conforms to the ethical guidelines of the Declaration of Helsinki as reflected in a prior approval by the Institutional Review Board of Lurie Children’s Hospital of Chicago. All methods were conducted in accordance with the Institutional Review Board’s guidelines and regulations.

### Macrophage quantification by immunohistochemistry

We performed immunohistochemistry to CD68 (Dako M0876), a cell surface marker on macrophages in normal, BA, and ALGS patients to determine if the hepatic macrophage population is expanded in cholestatic liver disease. De-waxing and antigen retrieval were performed on formalin-fixed, paraffin-embedded tissue sections following the Leica Bond-Max automated protocols. Image capture was performed with a 40x objective (400x) on a Nikon 80i (Nikon, Melville, NY, USA) microscope with a DS-Ri2 color camera. Images were stitched with a Prior Proscan III (Rockland, MA, USA) 8-slide stage and digital encoder, which allows capture of the entire tissue biopsy to a single image. The surface area was determined, and cell quantification was calculated with a thresholding algorithm on RBG images using NIS-elements AR (version 5.1). The same algorithm was utilized for all specimens, and the investigator who performed the quantification was blinded to disease classification. We performed a pairwise comparison between control and cholestatic groups and determined the level of significance by unpaired t-test.

### Human liver tissue digestion

We obtained fresh liver samples from explanted liver tissue for iBA, BASM, ALGS, and non-cholestatic pediatric liver (NC) at the time of transplantation. To account for variable disease throughout the liver, three 1 cm^3^ samples per patient were obtained from different areas of the explant by the clinical pathology team. Non-cholestatic liver tissue was taken from a patient with a hepatic neoplasm distal to the site of tumor. Matched histology slides from the explant were prepared by random sampling simultaneously from the explant by the pathology technician. Samples were stored in Tissue Storage Solution (Miltenyi Biotec) for 6 hours prior to mechanical and chemical digestion. Each of the three 1 cm^3^ samples per patient were split into thirds and infused with RPMI. We cut each sample into small pieces in a c-tube and added 2.5 mL digestion buffer per c-tube: 2 mg of DNase I (Sigma), 585 μL of Liberase TL (Sigma), and 9.215 mL of RPMI-1640 (Sigma R8758). Liver tissue was further digested using a both the Miltenyi Biotec gentleMACS Dissociator and incubation with shaking at 37°C for 1 hour. We strained the liver homogenate through a 40 μm filter into a 50 mL conical tube with grinding and washing to optimize yield. The samples were spun at 300 rcf for 10 min (4°C), the supernatant was aspirated, and the pellet was resuspended in Pharm Lyse for 1 minute to lyse remaining red blood cells. The reaction was stopped with HBSS, the samples were spun again at 300 rcf for 7 min (4°C), and the supernatant was aspirated. We resuspended the pellet in HBSS and strained twice over a 40 μm filter into a 15 mL tube. Cell count was performed and the cell suspension was prepared for fluorescence activated cell sorting (FACS).

### Flow cytometry and scRNA-seq library construction

A total of 1.9 x 10^7^ cells were obtained from digestion of ALGS liver, 2.2 x 10^7^ from BASM, 4.92 x 10^7^ from iBA, and 1.1 x 10^8^ from CL. We stained single cell suspensions from each sample with antibodies to detect cell viability and expression of the CD45 common leukocyte antigen. 90–100,000 live CD45+ cells were collected by fluorescence activated cell sorting with a viability of 94% for ALGS, 84% for BASM, 76% for iBA, and 87% for NC (S1 Fig). scRNA-seq libraries were prepared using the Single Cell 3’ v2 Reagent Kit for BASM and ALGS and the v3 Reagent Kit for iBA and NC (S2 Fig). Gel Beads in Emulsion containing single cells were generated by the 10x Genomics Chromium Controller in the Northwestern Next Generation Sequencing Facility. Barcoded libraries were sequenced on the Illumina HiSeq 4000 platform. Raw sequence data was processed using the 10X Genomics Cell Ranger 3.1.0 pipeline for de-multiplexing, trimming, aligning, and mapping to genes. After filtering of the scRNA-seq data 5,027 immune cells in ALGS, 2,633 immune cells in BASM, 5,927 immune cells in iBA, and 4,691 immune cells in NC were detected (S2 Fig).

### Single-cell RNA-seq analysis

To define the hepatic immune cell heterogeneity, we analyzed each single cell library using the Seurat version 3.0 R toolkit [22, 23]. Filtering parameters for each sample were set to include genes expressed in > 3 cells. Cells were included with gene counts >200 and < 5000, and with < 20% mitochondrial genome. We next ran the functions LogNormalize (scale factor 10,000), ScaleData, and RunPCA on each dataset. Variability in each principal component was visualized by the ElbowPlot function (S2C Fig). Based on this analysis we clustered the cells by the FindNeighbors function (15 dimensions for ALGS, 17 for BASM, 12 for iBA, and 10 for NC) and FindClusters (resolution of 0.5 for each cholestatic sample and 0.2 for NC). Cell clusters were visualized by Uniform Manifold Approximation and Projection (UMAP) using the function RunUMAP. Using lineage-specific marker genes, we annotated each cluster as myeloid (*CD68*, *CEBPB*, *CLEC9A*), T and natural killer (NK) cells (*CD3D*, *CD8A*, *NKG7*), and B cells (*CD79A* without *MZB1*) plasma cells (*CD79A* co-expressed with *MZB1*), and dividing cells (*TOP2A*). To confirm our cell assignments we used SingleR [24] to compare all clusters from each patient to the reference bulk transcriptome data from Immgen [25]. We also separately compared our disease-specific myeloid clusters to the Immgen database to further refine our myeloid subset annotations prior to integrated analysis. We next performed integrated clustering on the mononuclear phagocyte cells from each cholestatic patient and ran FindIntegrationAnchors and IntegrateData on ALGS clusters 5, 8, 10, BASM clusters 0, 6, 7, and iBA 3, 6, 9, 11, and 12. We determined the conserved genes within each integrated myeloid cluster by the function FindConservedMarkers. To compare our diseased macrophages to normal macrophages, we imported previously published single-cell data on non-diseased adult human liver from 5 donors with a median age of 41.0 years (interquartile range 23.5 to 54.5 years) [21]. We used the same cell-specific annotations and assigned this normal data-set as the reference in further SingleR analysis of our cholestatic macrophages. The degree of similarity between groups was further assessed visually by UMAP and by correlation analysis of shared genes (Morpheus, https://software.broadinstitute.org/morpheus). To infer pseudotime values, we used Monocle 3 [26–29] for trajectory analysis of non-diseased macrophages. By grouping cells into 5 clusters based on their pseudotime values, we applied the function FindGeneModules to identify 2 gene modules upregulated at the beginning (pseudotime 0–5) and end (pseudotime [20–25] of the trajectory to best represent the non-inflammatory and inflammatory macrophage profiles, respectively. Finally, to ascertain if differences in the transcriptional signatures may be secondary to patient age, we compared macrophages from the NC liver sample to the adult normal macrophages by correlation and pseudotime analyses as described above.

### Immunofluorescence and quantification of macrophage subsets

We next characterized protein expression for genes that differentiated the 3 cholestatic macrophage subsets by immunofluorescence using the Vectra Multispectral Imager in the Northwestern Immunotherapy Assessment Core. Baking and dewaxing was performed on formalin-fixed, paraffin-embedded tissue sections. Using the Opal 7-color automation kit (Akoya Biosciences, Marlborough, MA, USA) slides were stained for CD68 (Abcam ab955), CD69 (Abcam ab233396), C1Q (Abcam ab268120), and S100A8/9 (Abcam ab22506). Whole slide fluorescent imaging was performed followed by multispectral imaging of three 2.01 mm x 1.5 mm areas per slide (Phenochart and Vectra software). We next used inForm software to phenotype the cells and analyzed the cell data with phenoptrReports 0.2.9 package in R. Based on gene expression data we defined LAM on histology as CD68^+^C1Q^+^S100A8/9^-^CD69^-^, MLM as CD68^+^C1Q^-^S100A8/9^+^CD69^-^, and AM as CD68^+^C1Q^+/-^S100A8/9^-^CD69^+^. Using these definitions, we compared abundance on histology by disease group.

## Results

### Increased macrophage numbers in obstructive cholestasis as compared to healthy liver controls

**Fig 1:**
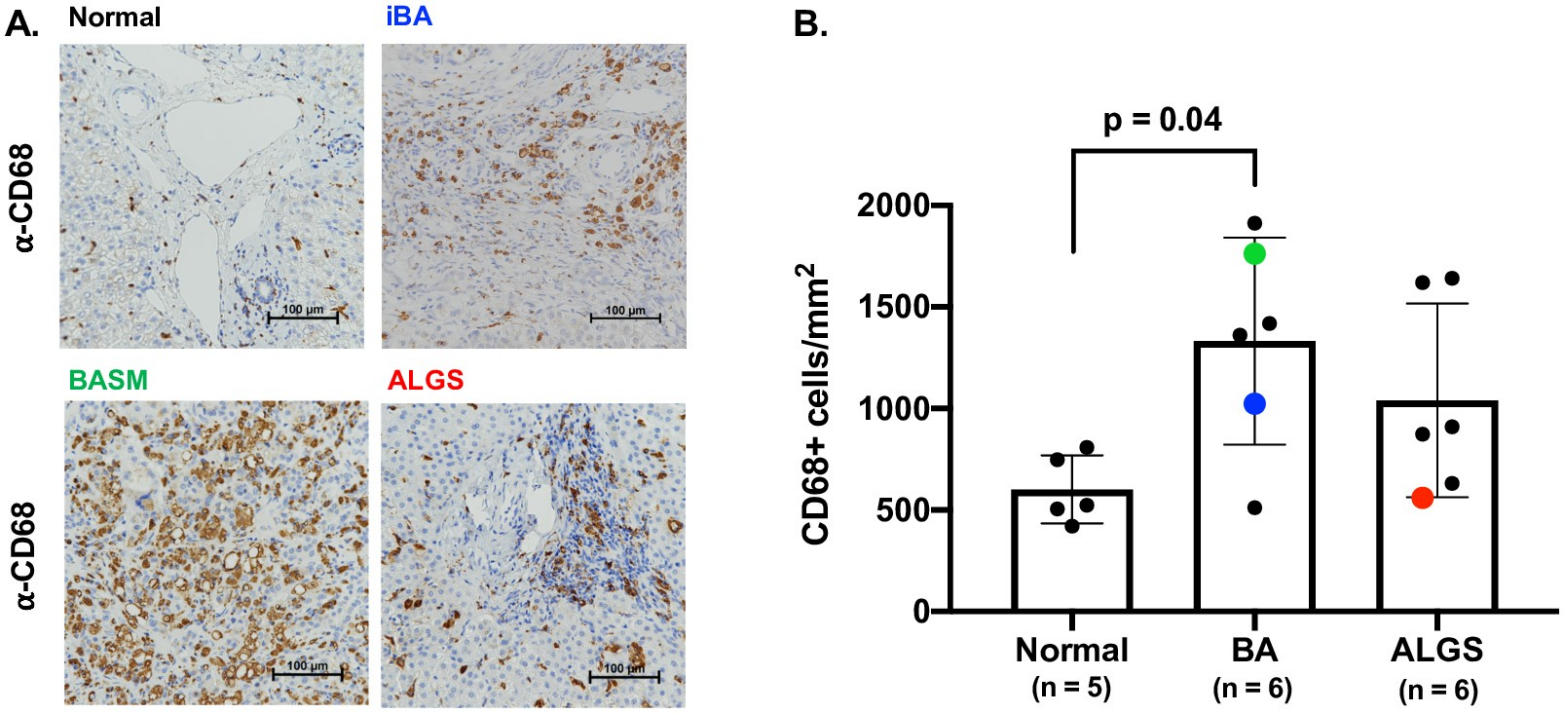
Increased hepatic macrophages in cholestatic liver disease. Representative immunohistochemistry staining with the macrophage marker anti-CD68 in samples taken at the time of liver transplantation from the iBA, BASM, and ALGS patients also used for scRNA-seq are shown compared to a normal donor liver sample (A). Quantitative analysis of entire sections from wedge biopsies showed a significantly increased number of CD68+ macrophages in BA patients, with individual samples processed for scRNA-seq shown in blue (iBA), green (BASM) and red (ALGS) (B).

We performed immunohistochemistry on histology samples from donor livers, and BA and ALGS patient livers at the time of liver transplantation to determine whether the hepatic macrophage population is expanded in cholestatic liver disease (Fig 1A). No histologic abnormality was present among donors with the exception of one individual liver which exhibited hepatocyte swelling. Mean age for donor pediatric patients rounded down to the nearest month was 68 months (SD 113, n = 5). No laboratory data was available for donor controls. All BA and ALGS liver samples had prominent fibrosis or cirrhosis at the time of tissue collection. Mean age rounded down to the nearest month for BA patients was 7 months (SD 1, n = 6) and 105 months (SD 78, n = 6) or 8 years and 9 months for ALGS cases. Difference in age between the 3 groups was not statistically significant by ANOVA (p = 0.12). Mean direct bilirubin within 24 hours prior to transplant was not significantly different between disease groups at 9.6 mg/dL (SD 8.0, n = 6) for BA and 12 mg/dL (SD 7.7, n = 6) for ALGS (p = 0.65 by paired t-test). We found increased number of CD68^+^ macrophages in BA liver as compared to control with a mean of 1332 cells/mm^2^ in BA versus 601 cells/mm^2^ in non-diseased pediatric liver tissue (p = 0.04) (Fig 1B). While ALGS samples also exhibited greater numbers (1040 cells/mm^2^) of CD68^+^ macrophages, it did not reach significance compared to control (Fig 1B). The pronounced influx of macrophages in cholestatic liver disease suggests they may play a pathogenic role in cholestatic-induced liver injury.

### Clinical information

We obtained liver tissue at the time of transplantation from three pediatric patients with cholestatic liver disease. Cholestasis with variable elevation of liver enzymes was present at the time of tissue collection for the iBA, BASM, and ALGS patient samples based on laboratory data close to the time of sample collection (S2A Fig). The iBA case was female and presented at nearly 6 months of age with a diagnosis of BA and did not receive a Kasai Portoenterostomy. She developed cirrhosis and portal hypertension and was thereby evaluated for primary liver transplantation. The patient with BASM was also female and received a late diagnosis of BA without Kasai Portoenterostomy. She developed cirrhosis and had a pre-transplant course complicated by portal hypertension, liver synthetic dysfunction, and infection. She received a transplant at 6 months of age. The patient with ALGS was male and met criteria for liver transplantation due to refractory fat-soluble vitamin deficiency leading to severe hepatic osteodystrophy and malnutrition. He had preserved liver synthetic function and while he did not have clinically evident portal hypertension at the time of transplant at 22 months of age he had findings of mild to moderate portal fibrosis with numerous bridges on histology.

### Variable immune cell composition between BASM and ALGS

**Fig 2:**
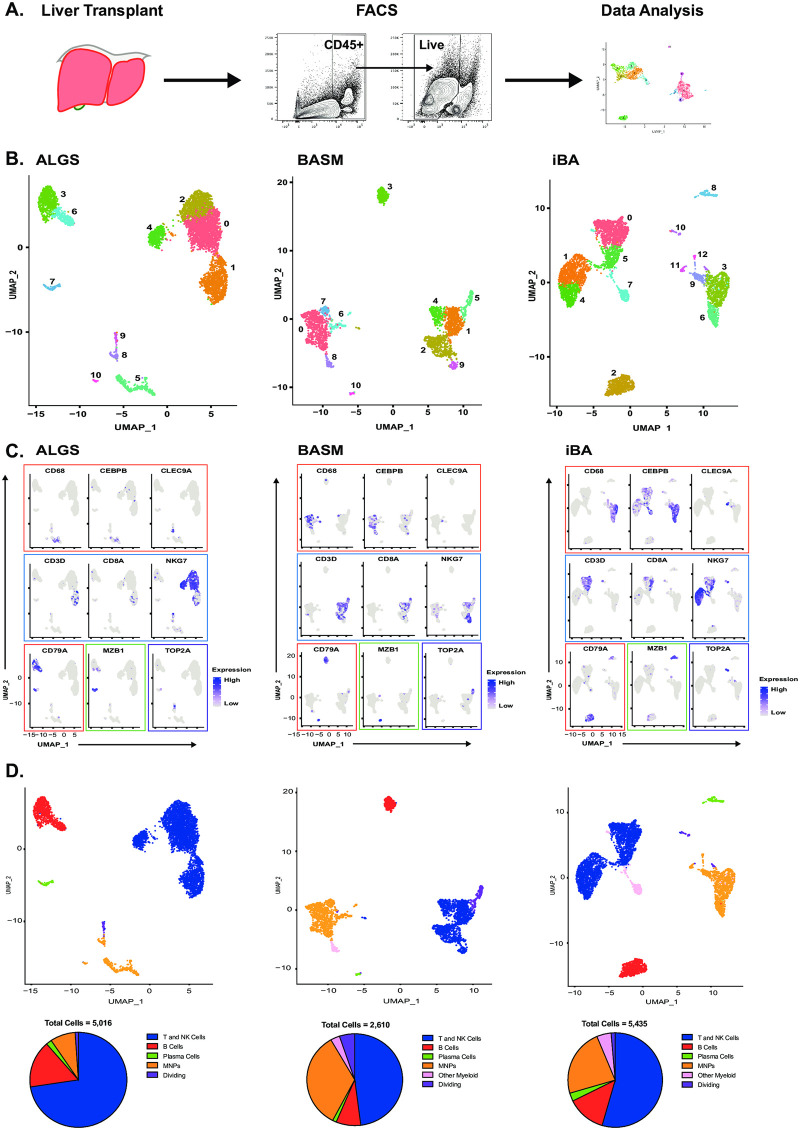
Single-cell RNA-seq enables immune cell characterization in cholestatic liver disease. Hepatic CD45+ cells were isolated from liver tissue at the time of liver transplantation by fluorescence activated cell sorting (FACS) for single-cell RNA-seq (scRNA-seq) analysis (A). UMAP of scRNA-seq data showing 11 clusters in ALGS (left) and BASM (middle) and 13 clusters in iBA (right) patient samples (B). Clusters were assigned to cell types based on the expression of lineage-specific genes (blue = T/NK cells; red = B cells; green = plasma cells; orange = MNP; pink = other myeloid cells; purple = dividing cells) in ALGS, BASM, and iBA (left to right) (C). UMAPs were re-colored by cell type and proportion of immune cells demonstrates greater numbers of MNP cells in BASM (middle) as compared to ALGS (right) and iBA (right) (D).

We next performed scRNA-seq on CD45^+^ live cells isolated from each liver sample to better evaluate immune cell infiltration in obstructive cholestasis (Fig 2A). We classified single-cell clusters into 5 immune cell types and a population of dividing cells in the cholestatic liver samples using lineage-specific marker genes (Fig 2B and 2C). Different clusters of the same cell type were highly correlated within each sample and between the samples thereby supporting the lineage annotation (S3A and S3B Fig). Further, the lineage annotations were confirmed by Single-R [24], which compares each cell against a reference dataset of population-level transcriptional profiles (in this case, the Immgen database [25]) (S3C Fig). Lastly, one cluster in each patient expressed high levels of cell cycle genes [30], which would indicate dividing cells (S3D Fig). T and NK cells were the most abundant immune cell population in all samples, comprising 73%, 48%, and 54% of total immune cells in ALGS, BASM, and iBA respectively (Fig 2D). Mononuclear phagocytes were the next largest population in BASM and iBA, but not in ALGS. This discrepancy may reflect the difference in disease etiology.

### Three distinct macrophage populations in obstructive cholestasis

**Fig 3:**
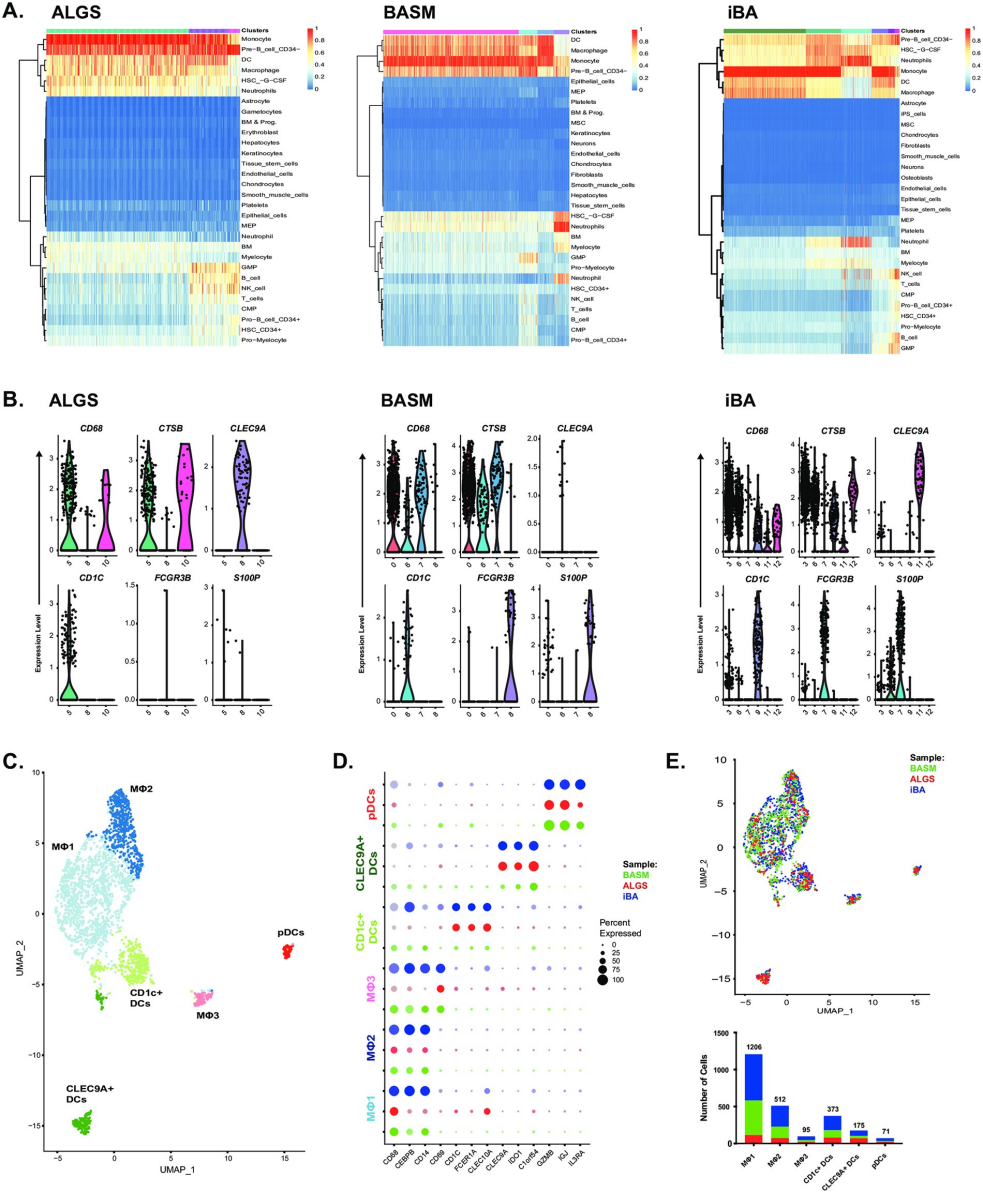
Integrated analysis of myeloid cells across patients identifies 3 distinct macrophage subsets in cholestasis. Comparison of annotated ALGS (5, 8, and 10, left), BASM (0, 6, 7, and 8, middle) and iBA (3, 6, 7, 9, 11, and 12, right) myeloid cell clusters with reference data set identified neutrophil clusters in BASM (8) and iBA (7) (A). While all other clusters expressed macrophage and/or dendritic cell markers, BASM cluster 8 and iBA cluster 7 expressed neutrophil markers *FCGR3B* and *S100P* (B). UMAP of remaining MNP cells showed 6 integrated clusters (C). Expression of key markers enabled identification of CD1c+ DCs (light green), CLEC9A+ DCs (dark green), pDCs (red), and 3 macrophage subsets (light blue, dark blue, and pink) across ALGS (red), BASM (green), and iBA (blue) patients (D). BASM (green) and iBA (blue) cells represented the majority in the macrophage clusters (E).

To better understand macrophage heterogeneity in obstructive cholestasis, we focused our analysis on the clusters annotated as MNP and other myeloid cells. Our Single-R results suggested these clusters contained a mixture of macrophages, dendritic cells (DCs), and neutrophils (Fig 3A). For further analysis, we excluded neutrophils, which were found in BASM (cluster 8) and iBA (cluster 7) and defined by distinct expression of neutrophil genes, such as *FCGR3B* and *S100P* [31], and lack expression of macrophage genes, such as *CD68* and *CTSB* (Fig 3B). We then performed integrated clustering on the remaining cells from all patients to define 3 macrophage subsets and 3 dendritic cell subsets (Fig 3C and 3D, S4 Fig). Three macrophage clusters were identified by the lineage-specific markers *CD68*, *CEBPB*, *CD14*, and *CD69*, (Fig 3C and 3D). The dendritic cells were annotated using markers described previously [32] to identify a *CD1C* positive subset, *CLEC9A* positive subset, and plasmacytoid DC (pDC) subset (Fig 3C and 3D). All macrophage populations were represented in each patient (Fig 3E, S4 Fig). Together, these findings suggest common macrophage subsets may arise from environmental cues in the setting of cholestasis.

**Fig 4:**
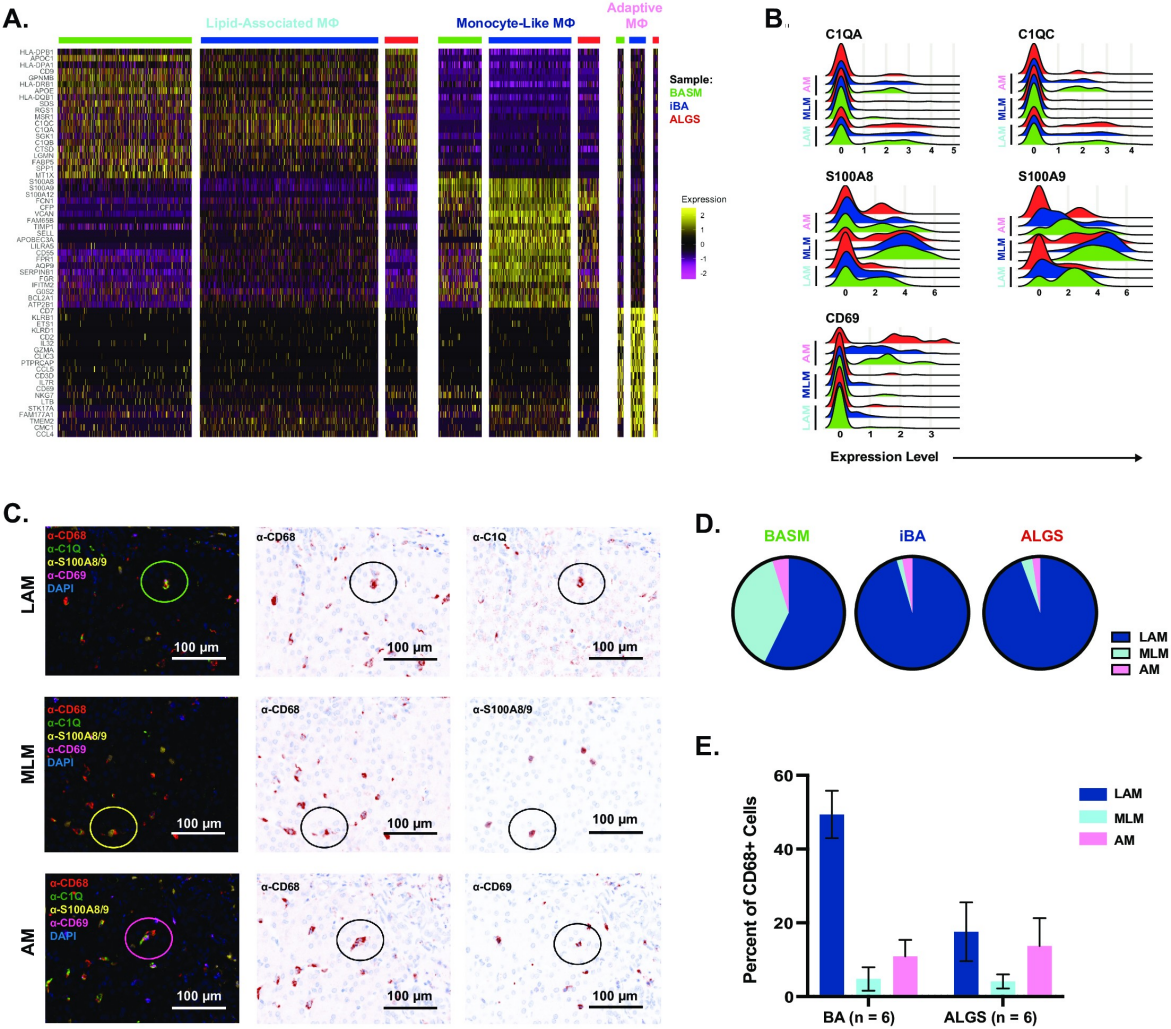
The transcriptional signature of macrophage subsets is conserved across patients. Each macrophage subset exhibited a unique transcriptional signature that was similar between ALGS (red), BASM (green), and iBA (blue) patients (A). Ridge plot demonstrates the expression of genes upregulated in each subset including *C1QC* and *C1QA* in lipid-associated macrophages, *S100A8* and *S100A9* in monocyte-like macrophages, and *CD69* in adaptive macrophages (B). Representative images of immunofluorescence identifying co-localization of each protein marker with anti-CD68 to identify LAM, MLM, and AM(C). The relative contribution of each subset to total cholestatic macrophages was compared between the BASM, iBA and ALGS patients with corresponding scRNA-seq data (left to right) (D). Average percent of total CD68+ cells with standard error of mean for each macrophage subset in 6 BA and 6 ALGS patients with prior CD68 quantification (E).

We next sought to characterize the cross-disease transcriptional signature of each inflammatory macrophage subset and defined MΦ1 as lipid-associated macrophages (LAM), MΦ2 as monocyte-like macrophages (MLM), and MΦ3 as adaptive macrophages (AM) (Fig 4A and 4B). LAM demonstrated the highest expression of genes associated with lipid metabolism including *APOC1*, *APOE*, *LGMN*, *FABP5*. There was also high overlap with genes previously reported in LAM from human adipose tissue including *TREM2* (S5 Fig) [33]. MLM were defined by genes previously identified in monocytes, including *S100A8*, *S100A9*, *VCAN* [34–36]. Finally, AM were enriched for genes associated with lymphocyte activation including *CD2*, *CD7*, *CCL5*, *CCL4*, *CD3D*, *IL7R*. As we have previously defined these immune cells as macrophages, the increased expression of genes involved in adaptive immunity suggest these cells may have engulfed lymphocytes or play a role in regulation of lymphocyte response.

To validate these three populations across cholestatic liver disease, we performed immunofluorescence on a large cohort of patients. We chose markers for each population based on their differential gene expression by scRNA-seq (Fig 4B). Using these markers, we demonstrated the presence of all subsets across the fixed BA and ALGS samples from Fig 1 through overlap with CD68 expression (Fig 4C). Since not all individual cells in a population expressed the relevant marker, we expect this approach to have lower sensitivity than specificity as supported by differences between histology and gene expression analyses for the BASM, iBA, and ALGS samples (Fig 4D). Thus, the percent of each population is likely to be an underestimate and may explain the proportion of CD68+ cells not assigned to any population. Despite these differences, comparing the number of cells in each population between 6 BA and 6 ALGS patients shows that LAM tends to account for a greater proportion of macrophages in BA (Fig 4E). In contrast, the AM population is larger on average in ALGS patients. Further study is required to determine whether this difference reflects disease pathogenesis.

### Reduced expression of immune-regulatory genes in obstructive cholestasis as compared to non-diseased human liver

**Fig 5:**
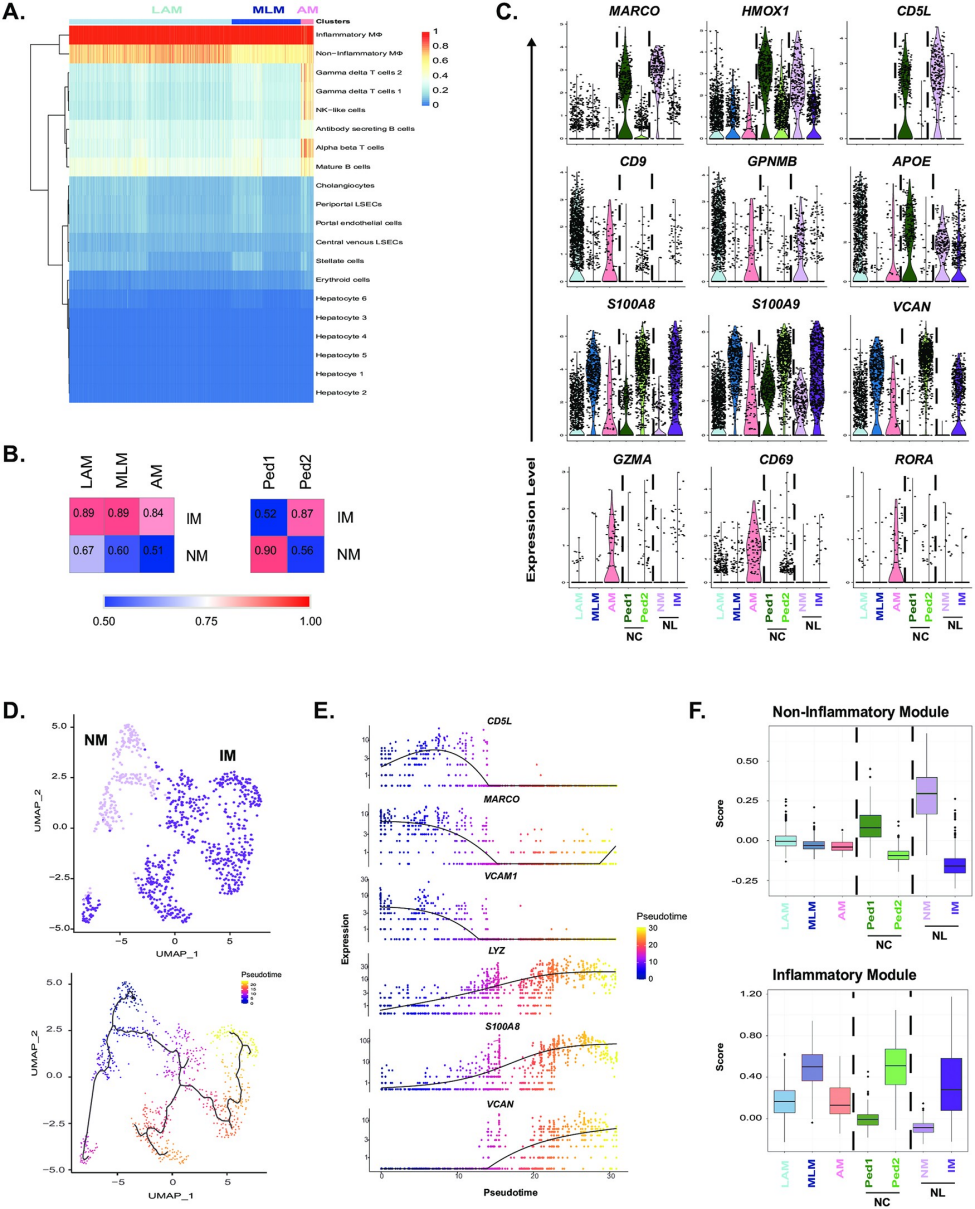
Cholestatic macrophages are distinct from non-diseased hepatic macrophages. All three cholestatic macrophage subsets were primarily assigned by SingleR to previously published inflammatory macrophages (IM) in non-diseased liver (A). The mean gene expression of each cholestatic macrophage subset was more highly correlated with inflammatory macrophages (IM) than non-inflammatory macrophages (NM) (left). Of the two macrophage subsets in pediatric non-cholestatic (NC) liver, Ped1 was most similar to NM, while Ped2 was similar to IM (right) (B). Violin plots of individual genes that define similarities and differences in expression between cholestatic macrophage subsets as compared to healthy adult and pediatric NC macrophages (C). Pseudotime analysis of healthy adult macrophages given a beginning (indicated with black circle) at NM (light purple) inferred a trajectory ending in IM (dark purple) (D). The non-inflammatory module contained genes with expression peaking at pseudotime 0–5, while the inflammatory module peaked at pseudotime 20–25 (E). The non-inflammatory module is lowly expressed across all cholestatic macrophages whereas MLM demonstrated high expression of the inflammatory module (F).

We took advantage of single-cell data that was previously published using non-diseased adult livers [21] to determine how macrophages from cholestatic livers compared to those from healthy livers. We reproduced the 20 clusters from the original study of which 2 were labelled as “inflammatory” (IM) and “non-inflammatory” (NM) macrophages (S6A Fig). Although this data included all cell types, not just CD45+ cells, annotation of immune cell types using lineage-specific markers led to analogous results (S6B Fig). To overcome technical variability between data-sets limiting the utility of co-clustering, we used Single-R, Correlation analysis, and single gene and gene set comparisons to evaluate similarities and differences between macrophage subsets. All 3 populations of cholestatic macrophages were more similar to the IM than NM (Fig 5A and 5B); AM was the least correlated overall (0.84) compared to LAM (0.89) and MLM (0.89) (Fig 5B).

To determine whether the differences between datasets was due to older age of the controls, we performed scRNA-seq on a pediatric non-cholestatic (NC) pediatric liver sample. The NC case was an 11-year-old female whose explanted liver demonstrated some areas of necrosis consistent with changes after chemotherapy and chronic inflammation with margins negative for tumor. Through a comparable scRNA-seq analysis work-flow, we identified two populations of macrophages, which we label Ped1 and Ped2 (Fig 5B and S7 Fig). Unlike the cholestatic macrophages, these populations clearly recapitulate the dichotomy of adult NM and IM (Fig 5B). Moreover, while all cholestatic macrophages demonstrated decreased expression of immunoregulatory genes (*MARCO*, *HMOX1*, and *CD5L*), Ped2 expressed these genes at comparable levels to NM (Fig 5C). The cholestatic populations, LAM and AM, exhibited distinct transcriptional signatures from both adult and pediatric macrophages subsets (Fig 5C). In contrast, the genes that defined MLM were also increased in adult IM and Ped2 (Fig 5C). Interestingly, expression of *NR1H4*, which encodes FXR and is thought to play a role in macrophage inflammasome activation in cholestasis, is negligible across all macrophages (S8 Fig). Taken together, our findings support the emergence of disease-specific macrophages in cholestasis that may mediate inflammation via different pathways than FXR signaling.

Although transcriptionally distinct, macrophages in the diseased liver may be derived from their healthy counterparts. Using Monocle, we defined a pseudotime trajectory beginning in NM (pseudotime 0) and ending in IM (pseudotime 25) (Fig 5D, S9A Fig). We then identified 2 modules associated with high expression at these endpoints: the non-inflammatory module included genes such as *CD5L*, *MARCO*, and *VCAM1* whereas the inflammatory module included *LYZ*, *S100A8*, and *VCAN* (Fig 5E, S9 Fig). In support of the limited effect of age on healthy macrophage heterogeneity, the former modules were highest in Ped1, while the latter was highest in Ped2. In contrast, we found that no cholestatic macrophage subset expressed high levels of the non-inflammatory module (Fig 5F). However, MLM exhibited high expression of the inflammatory module, possibly indicating a common origin with IM (Fig 5F). This analysis demonstrates the transcriptional variability across cholestatic macrophages beyond the dichotomy of healthy liver macrophages.

## Discussion

We are the first to perform scRNA-seq on pediatric cholestatic liver to define the macrophage transcriptional profile in obstructive cholestasis. Hepatic macrophages play a critical role in maintaining immune tolerance in the setting of persistent exposure to bacterial antigens from the intestine. Loss of this tolerogenic phenotype in the setting of inflammation may be of particular importance in ongoing hepatic injury in obstructive cholestasis. Here, we identify three populations of pathogenic macrophages independent of underlying etiology that may contribute to liver injury in obstructive cholestasis. No cholestatic macrophage subset was characterized by expression of immune regulatory genes as seen in normal adult NM and a subset of macrophages in non-cholestatic pediatric liver (Ped1). Our data suggest that tissue-resident macrophages, such as NM previously reported [21], may be absent or transcriptionally altered by the tissue microenvironment in cholestatic liver injury. Instead, all 3 cholestatic macrophage subsets were most similar to IM, which are likely to be monocyte-derived macrophages. In particular, the population of monocyte-like macrophages had the greatest upregulation of genes encoding the S100 proteins in addition to *TREM1*, known to amplify the innate immune response [37], suggesting this population may have recently infiltrated [38]. We also defined a subset of lipid-associated macrophages that had the highest expression of genes involved in TLR signaling (e.g. *GPNMB* [39], *MT1G* and *MT1X* [40, 41]). Lastly, we demonstrate the presence of a novel adaptive macrophage subset with increased *RORA* gene expression, which has been shown to promote anti-inflammatory polarization of hepatic macrophages in a murine model of nonalcoholic steatohepatitis [42] and a human monocyte cell line [43]. The transcriptional profiling of these distinct subsets may identify macrophage-specific targets to ultimately inhibit monocyte recruitment, block TLR-mediated macrophage activation, or re-program macrophages to an anti-inflammatory phenotype.

While macrophages have been implicated in immune-mediated hepatic injury from cholestasis [9–13, 15–20], the exact mechanism is not well known. Current medical therapies for cholestatic liver disease include FXR agonists, which in addition to regulating the bile acid pool may also inhibit macrophage inflammasome activation based on prior studies [13]. However, we demonstrate an absence of *NR1H4* encoding FXR in cholestatic macrophages despite evidence of TLR signaling and inflammasome activation. As macrophages play a role in cholestatic liver injury, this finding highlights the lack of current cell-specific immune-modulatory strategies and the need for a deeper understanding of the immune response to cholestasis.

LAM in our samples had a gene signature that was similar to recently published data on *TREM2+* lipid-associated macrophages in murine adipose tissue [33]. This gene signature was also similar to human hepatic macrophages during obesity and may represent a conserved response to loss of metabolic homeostasis [33]. As hypercholesterolemia is a common sequela of cholestasis, the *TREM2*+ LAM in our samples may arise in response to similar metabolic derangements. However, LAM in our study differed in that they had an overall inflammatory gene signature despite expression of *TREM2* previously shown to promote anti-inflammatory macrophage polarization [33, 44]. They also were identifiable by C1Q expression, which was similarly expressed by NM from the healthy adult dataset. It is possible that LAM arise from inflammatory activation of healthy tissue-resident macrophages. Targeting the *TREM2* molecular pathway may be an important therapeutic target to re-program hepatic macrophages to an immune regulatory phenotype and reduce the consequences of hypercholesterolemia in cholestasis.

The mechanism of disease pathogenesis in BA is hypothesized to be multifactorial, including an aberrant immune response to a cognate antigen [45] whereas ALGS is a genetic disease resulting in bile duct paucity. Thus, while the aim of the current study was to identify a common cholestatic macrophage phenotype, there are likely etiology-specific differences in the immune pathways for macrophage activation that require further investigation in larger studies. However, despite this limitation, we provide important insight into hepatic macrophage heterogeneity in cholestatic liver disease compared to healthy livers. Despite age differences, it is worth noting that macrophages from a non-cholestatic pediatric patient demonstrated a similar dichotomy as adult hepatic macrophages. This finding suggests that the distinct transcriptional signature of cholestatic macrophages is not a result of age-specific differences or technical differences between datasets such as variation in sample isolation, processing and digestion protocols, or experimental design. Lastly, we acknowledge that our findings may not be limited to obstructive cholestasis and may overlap with other causes of end-stage liver disease characterized by cirrhosis and portal hypertension. A recent study on adult cirrhotic livers described a population of scar-associated macrophages in cirrhosis [46] that appear most similar to cholestatic LAM and express higher levels of *TREM2*, *CD9*, *LGALS3*, and *SPP1*. Future studies will more clearly define the similarities and differences in subset-specific macrophage function by patient age, stage of cholestatic liver disease, and etiology of cirrhosis.

In this study, we have used single-cell transcriptional analysis of pediatric cholestatic liver samples to identify macrophage subsets at greater resolution than previously described [47]. With ongoing work, we will strengthen conclusions on the hepatic macrophage transcriptional signature in different cholestatic liver diseases and identify common therapeutic targets to reprogram macrophages and slow disease progression. More specifically, we highlight expression of the immune regulatory genes *RORA* and *TREM2* within these inflammatory subsets that may be potential therapeutic targets to ameliorate inflammatory injury in obstructive cholestasis. Future work to correlate our findings to the immune cell subsets present earlier in disease will provide important insight into cell-specific therapeutic strategies to improve prognosis shortly after disease onset. Identifying molecular targets to reprogram hepatic macrophages in cholestasis may also have therapeutic implications for other etiologies of liver diseases and reduce the medical burden of end-stage liver disease.

## Supporting information

S1 Fig Gating strategy for fluorescence activated cell sorting of CD45+ live cells from liver tissue of a patient with ALGS (A), BASM (B), iBA (C), and NC (D). (PDF)

S2 Fig Demographic, laboratory, and sequencing data is provided for each patient (A). Age was rounded down to the nearest month. Reported laboratory values were obtained within 24 hours prior to liver transplant. Admission bilirubin levels and INR obtained 1 week prior to liver transplant before initiation of fresh frozen plasma and renal replacement therapy are reported in parentheses for BASM. Distribution of gene counts, unique molecular identifier (UMI) counts, and percent mitochondrial genes per cell in ALGS, BASM, iBA and NC (left to right) are shown by cluster (B). The standard deviation associated with each principal component (PC) in the analysis of each single-cell RNA-seq dataset (C).
(PDF)

S3 Fig Pairwise Pearson’s correlation of average gene expression between each cluster in ALGS, (left), BASM (middle), and iBA (right) organized by cell type annotation (pink = other myeloid; orange = MNP; blue = T/NK cells; red = B cells; green = plasma cells; purple = dividing cells) (A). Clustering of cell types between patients by principal component analysis (B). Single-R analysis of clusters from ALGS (left), BASM (middle), and iBA (right) compared to Immgen database reference dataset confirmed our cell cluster assignments (C). Dividing cells were identified in each patient sample by expression of the cell cycle genes *CDK1*, *UBE2C*, and *TOP2A* (D).
(PDF)

S4 Fig Visualization of clusters from integrated analysis of myeloid cells on original UMAP from Fig 2 and the proportion of MNP cells for ALGS (A), BASM (B), and iBA (C). Pairwise Pearson’s correlation of average gene expression between integrated cholestatic myeloid clusters (D).
(PDF)

S5 Fig Violin plots showing expression of genes associated with lipid-associated macrophages in human adipose tissue are most highly expressed in lipid-associated macrophages from cholestatic livers [33]. (PDF)

S6 Fig UMAP reproducing the 20 clusters of cells, including inflammatory macrophages (IM, cluster 4) and non-inflammatory macrophages (NM, cluster 10), from previously published scRNA-seq of non-diseased livers [21] (A). Expression of lineage-specific genes verifies the identify of immune cells clusters (blue = T/NK cells; red = B cells; green = plasma cells; purple = dividing cells; orange = MNP). The UMAP is recolored by cell type and the proportion of immune cells is shown (B).
(PDF)

S7 Fig UMAP of scRNA-seq data from a pediatric non-cholestatic liver (NC) shows 10 clusters of cells (A). Feature plot demonstrates expression of lineage-specific genes by cell cluster (blue = T/NK cells; red = B cells; green = plasma cells; orange = MNP; pink-other myeloid cells; purple = dividing cells; gray = endothelial cells) (B). Comparison of gene expression across all myeloid cell clusters identifies cluster 3 as neutrophils expressing FCGR3B and S100P, CD1c+ dendritic cells as cluster 7, and cluster 1 and 4 as macrophage clusters (C). Single-R analysis using previously published data from adult normal livers as the reference [21] supports our cluster assignments with the addition of neutrophil and dividing cell clusters (D). Re-colored UMAP by cell type and proportion of immune cells demonstrates high numbers of MNP and T/NK cells with contribution of endothelial cells from possible contamination (E).
(PDF)

S8 Fig Violin plots showing expression of individual genes involved in inflammasome activation across cholestatic and non-cholestatic macrophage subsets. *NR1H4* encoding FXR was absent in cholestatic macrophages.
(PDF)

S9 Fig Macrophages from non-diseased liver (NL) were categorized into 5 groups based on their inferred pseudotime (A). From 23 modules of genes with pseudotime-associated expression, we chose module 4 with increased expression at pseudotime 0–5 to represent the non-inflammatory module and module 2 with increased expression at pseudotime 20–25 to represent the inflammatory module (B). Visualization of gene expression for these 2 modules in non-cholestatic pediatric liver macrophages shows that module 4 is upregulated in Ped1 macrophages similar to non-inflammatory adult macrophages and module 2 is upregulated in Ped2 macrophages similar to inflammatory adult macrophages (IM) (C). Comparison to cholestatic macrophages demonstrated low expression of the non-inflammatory module across all subsets whereas MLM demonstrated high expression of the inflammatory module (D).
(PDF)
