# Supplementary figures and images for: Transcriptional profiling of pediatric cholestatic livers identifies three distinct macrophage populations
Source: PLoS One. 2021 Jan 7;16(1):e0244743. doi: 10.1371/journal.pone.0244743 (PMC7790256; doi:10.1371/journal.pone.0244743)

A. ALGS

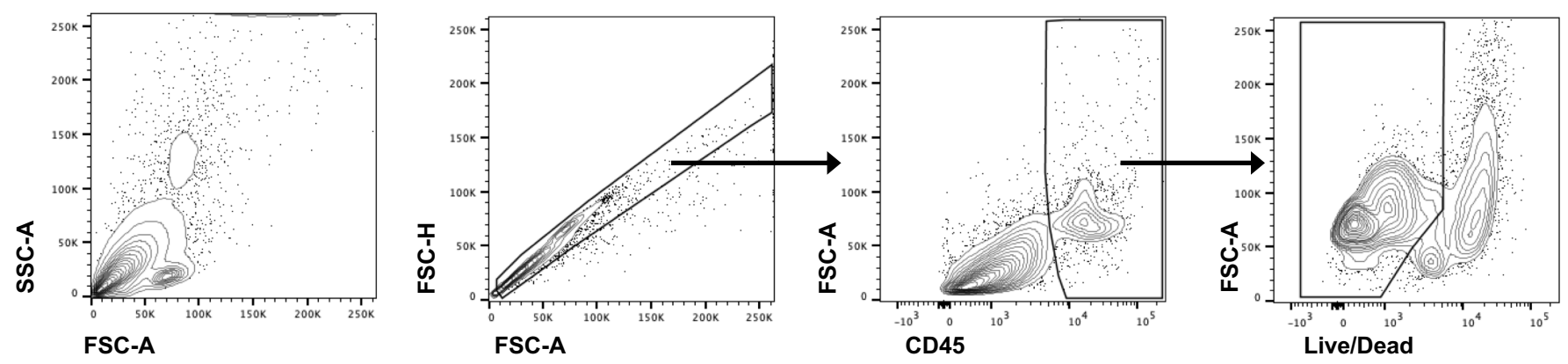

B. BASM

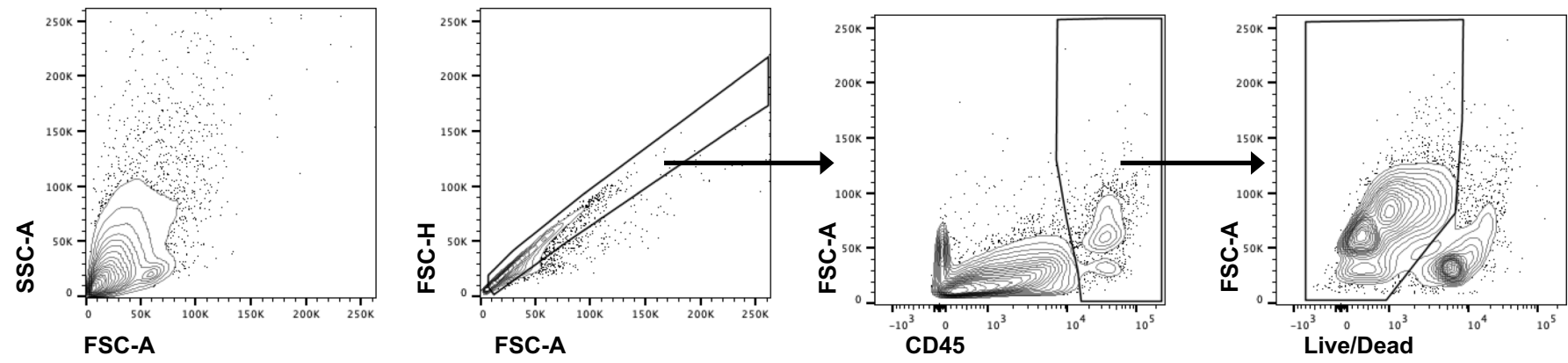

C. iBA

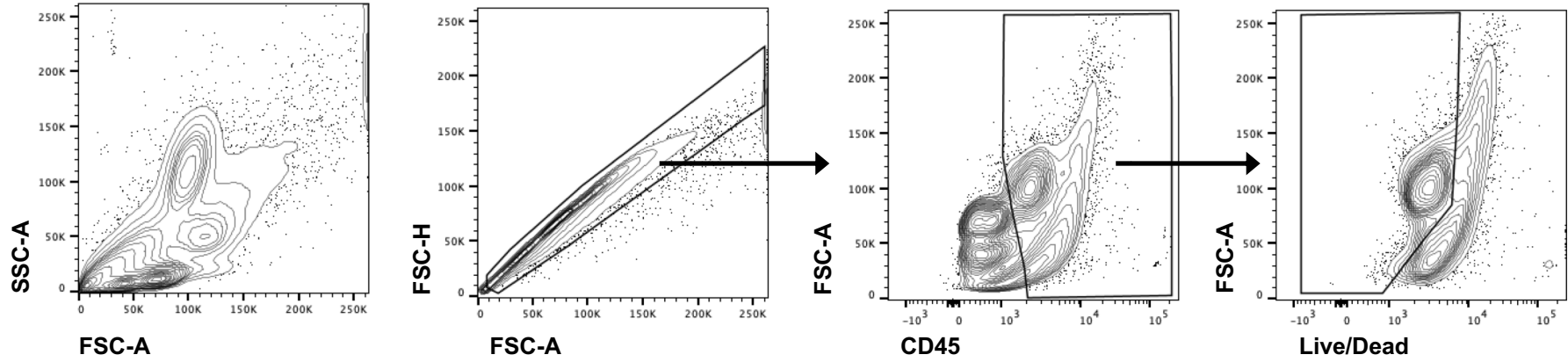

D. NC

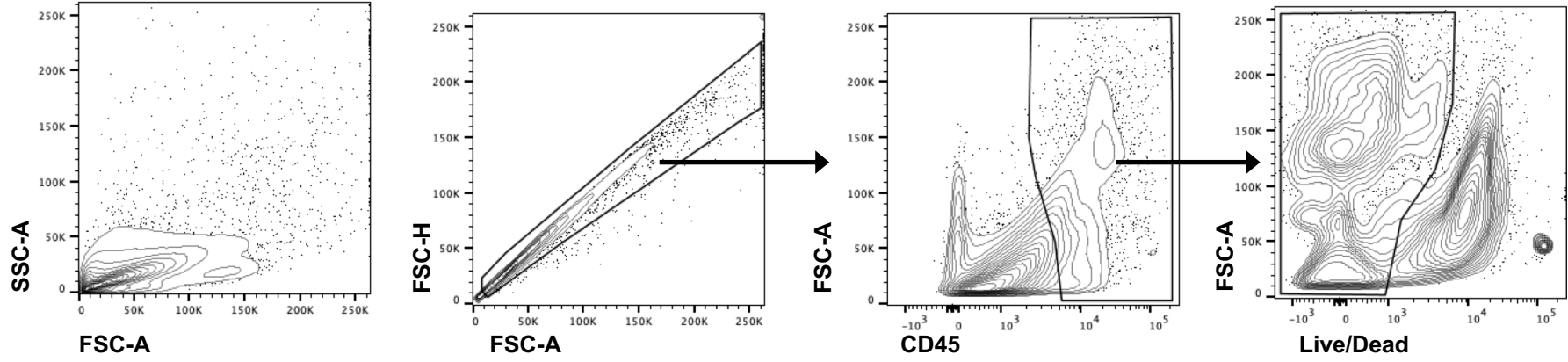

Supplement: S1 Fig — (PDF) [file pone.0244743.s001.pdf]

A. ALGS

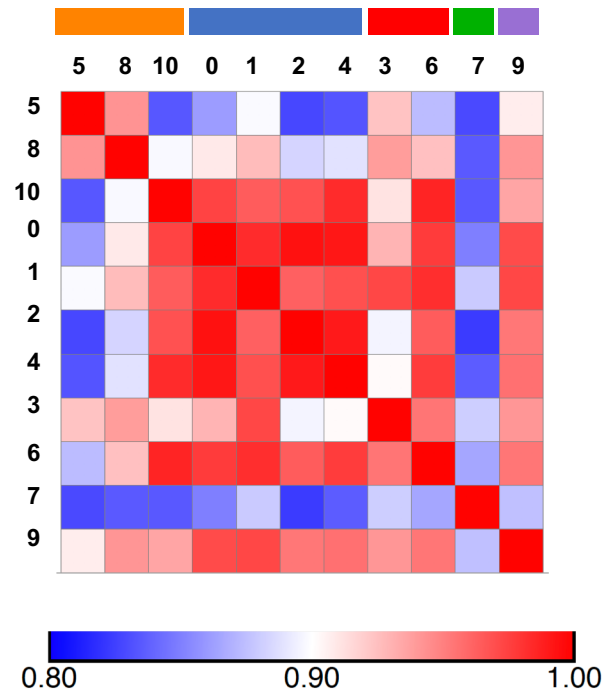

BASM

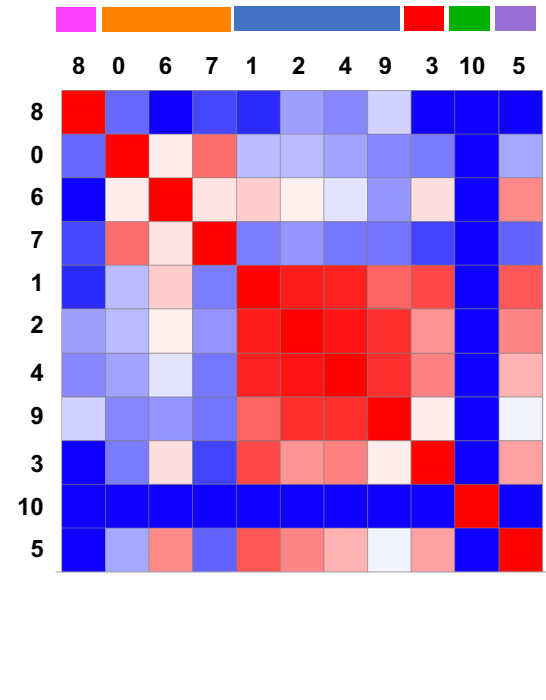

iBA

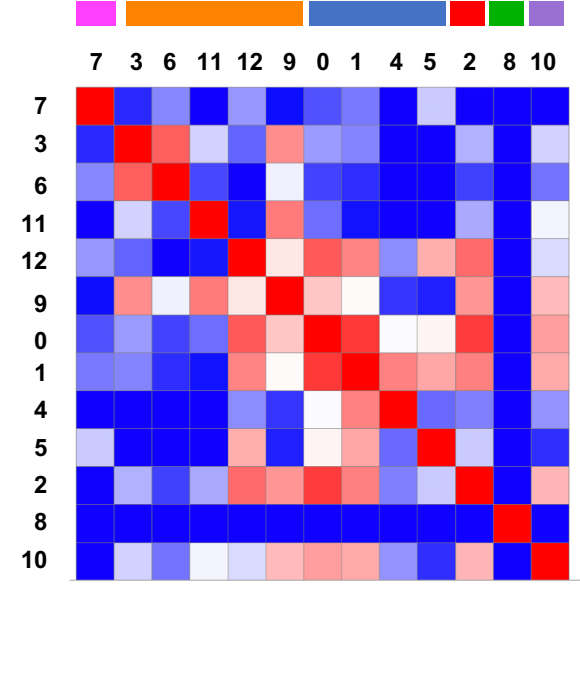

B.

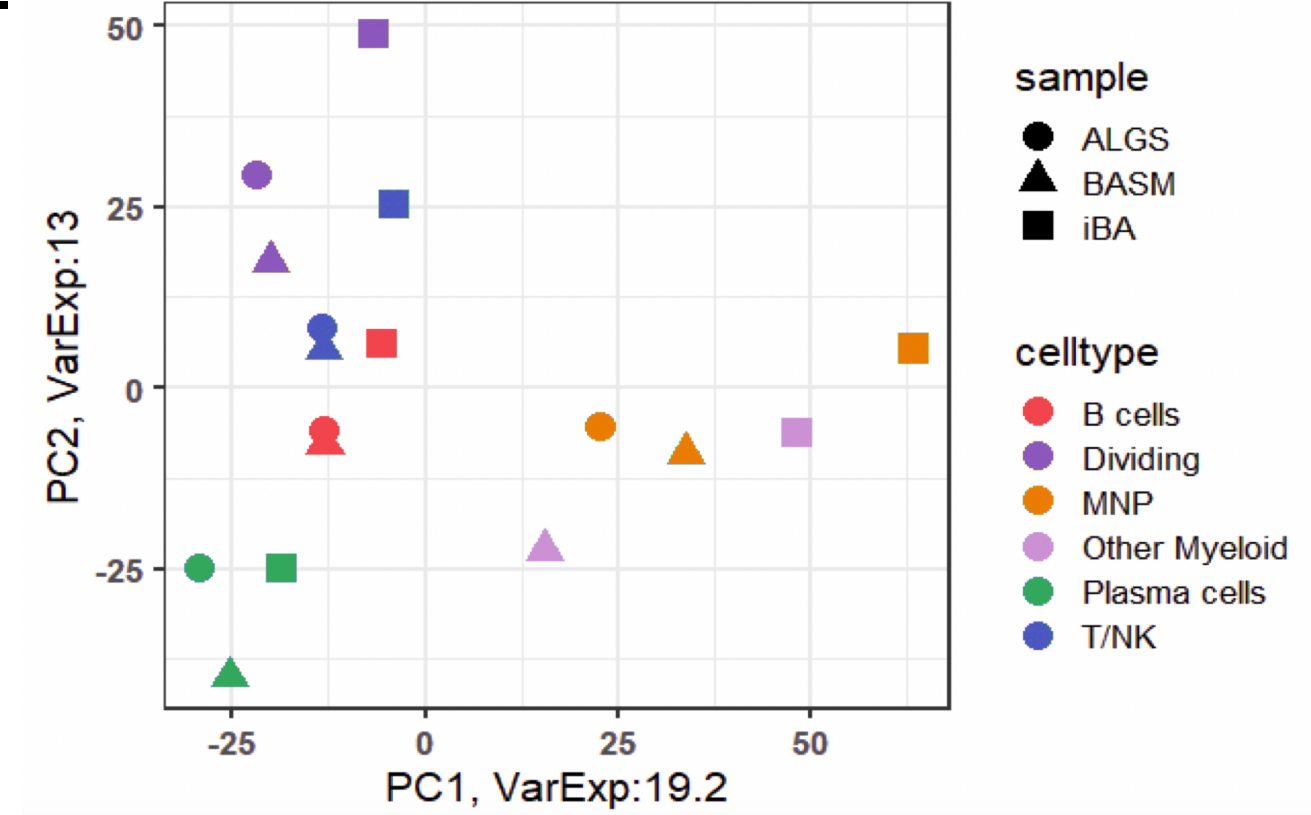

C. ALGS

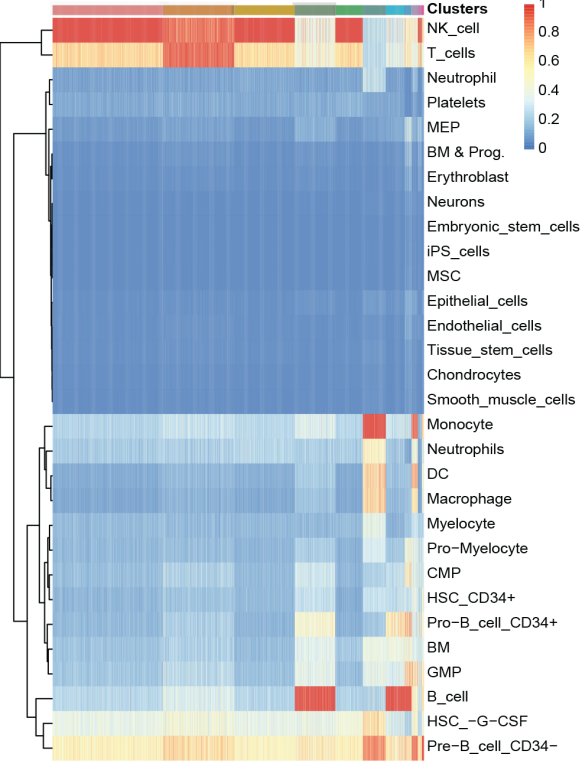

BASM

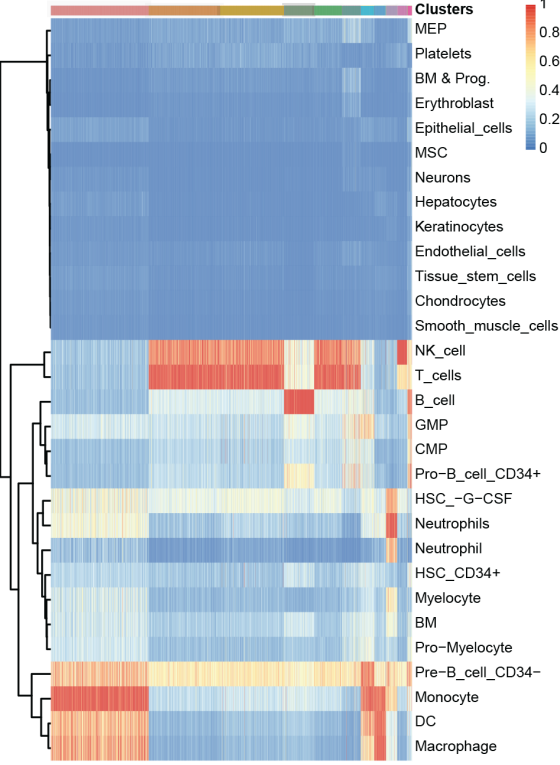

iBA

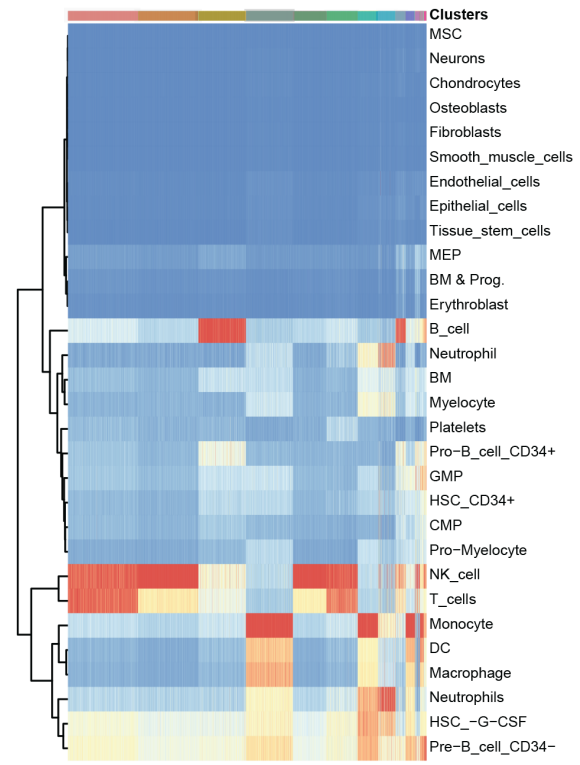

D. ALGS

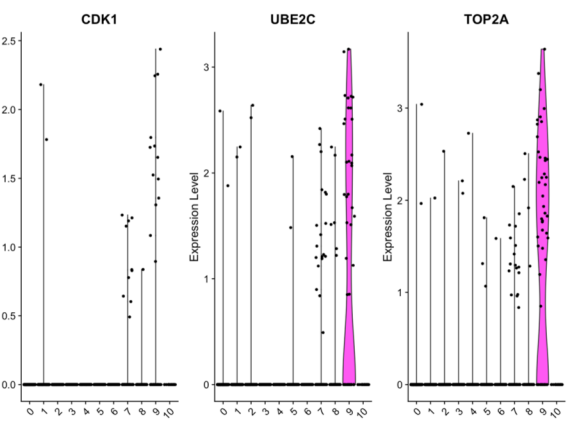

BASM

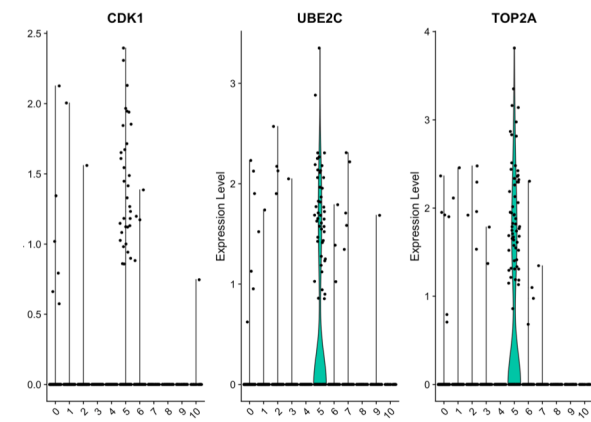

iBA

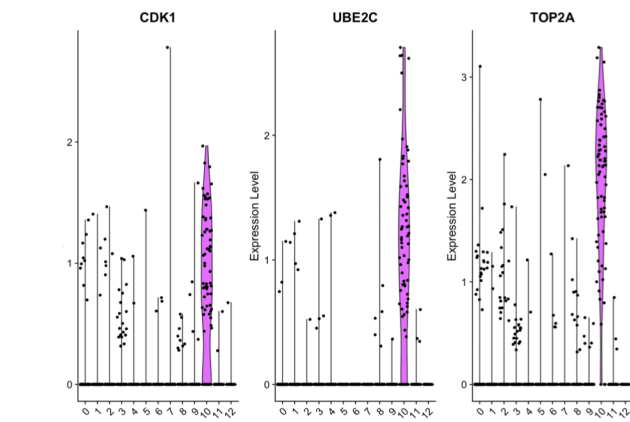

Supplement: S3 Fig — Pairwise Pearson’s correlation of average gene expression between each cluster in ALGS, (left), BASM (middle), and iBA (right) organized by cell type annotation (pink = other myeloid; orange = MNP; blue = T/NK cells; red = B cells; green = plasma cells; purple = dividing cells) (A). Clustering of cell types between patients by principal component analysis (B). Single-R analysis of clusters from ALGS (left), BASM (middle), and iBA (right) compared to Immgen database reference dataset confirmed our cell cluster assignments (C). Dividing cells were identified in each patient sample by expression of the cell cycle genes CDK1, UBE2C, and TOP2A (D). (PDF) [file pone.0244743.s003.pdf]

A. ALGS

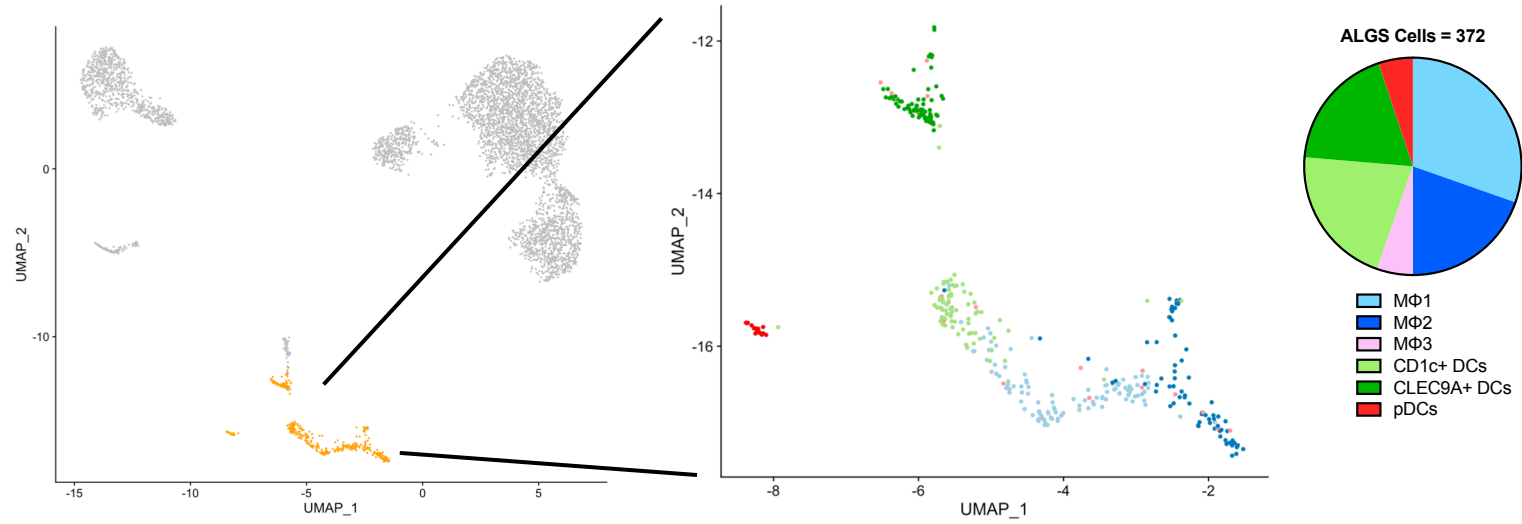

B. BASM

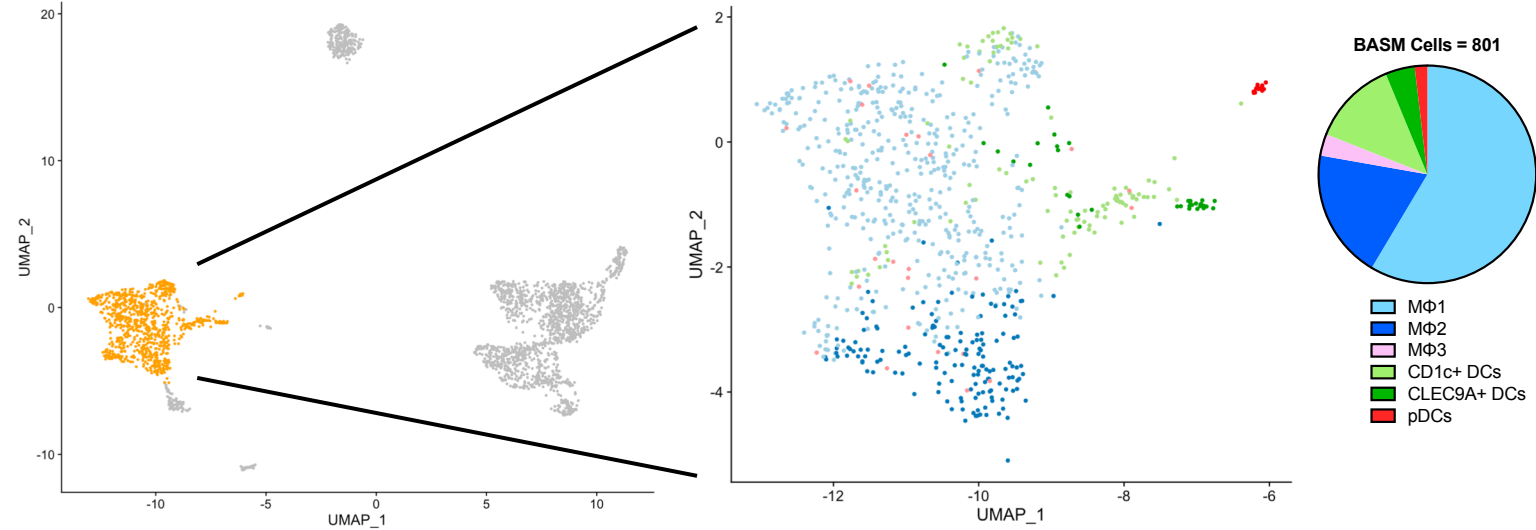

C. iBA

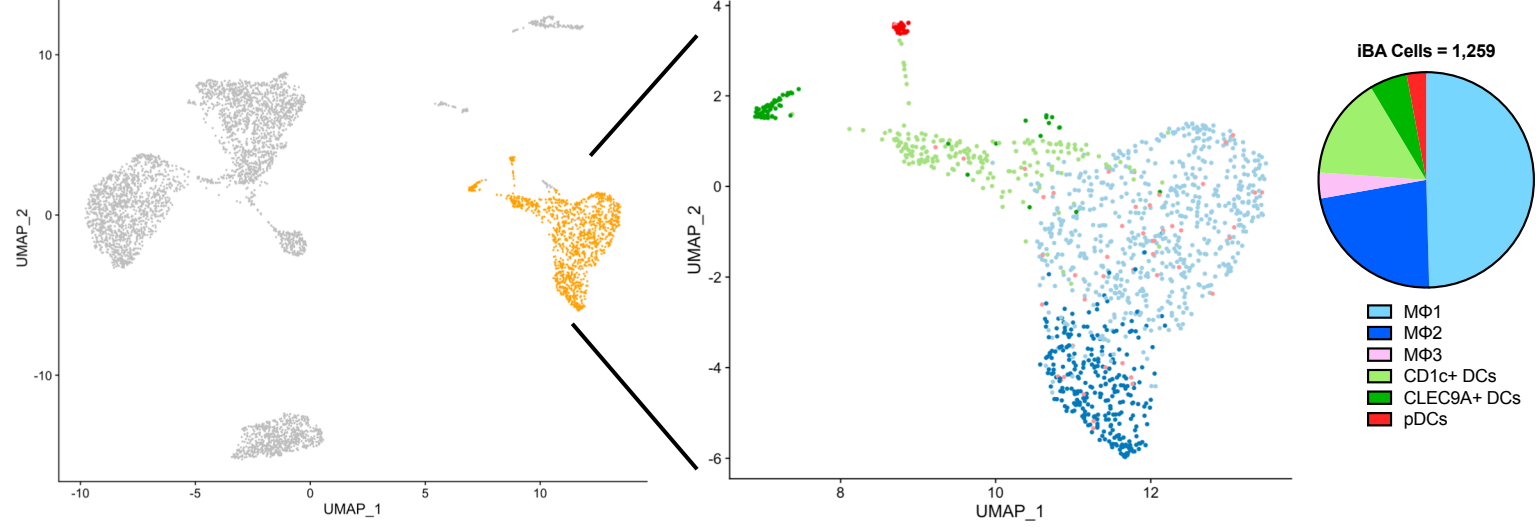

D. Cholestatic Myeloid

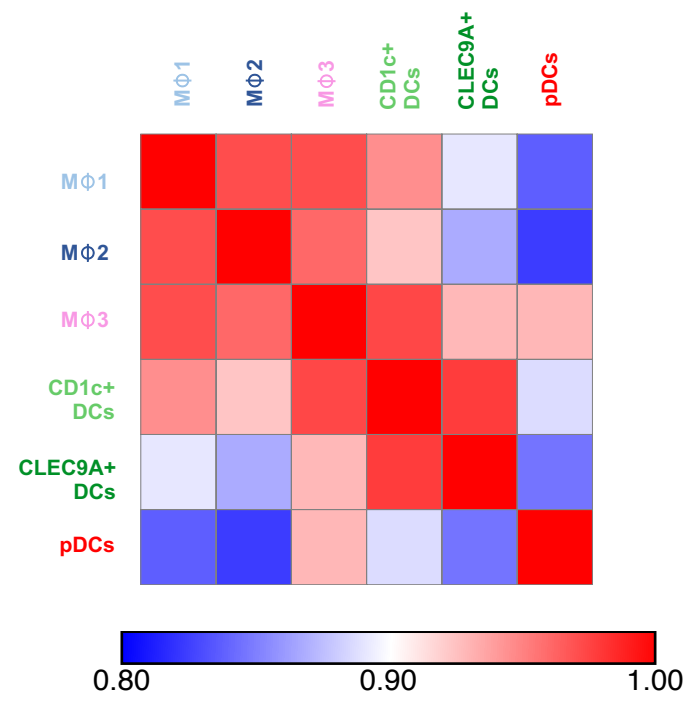

Supplement: S4 Fig — Visualization of clusters from integrated analysis of myeloid cells on original UMAP from Fig 2 and the proportion of MNP cells for ALGS (A), BASM (B), and iBA (C). Pairwise Pearson’s correlation of average gene expression between integrated cholestatic myeloid clusters (D). (PDF) [file pone.0244743.s004.pdf]

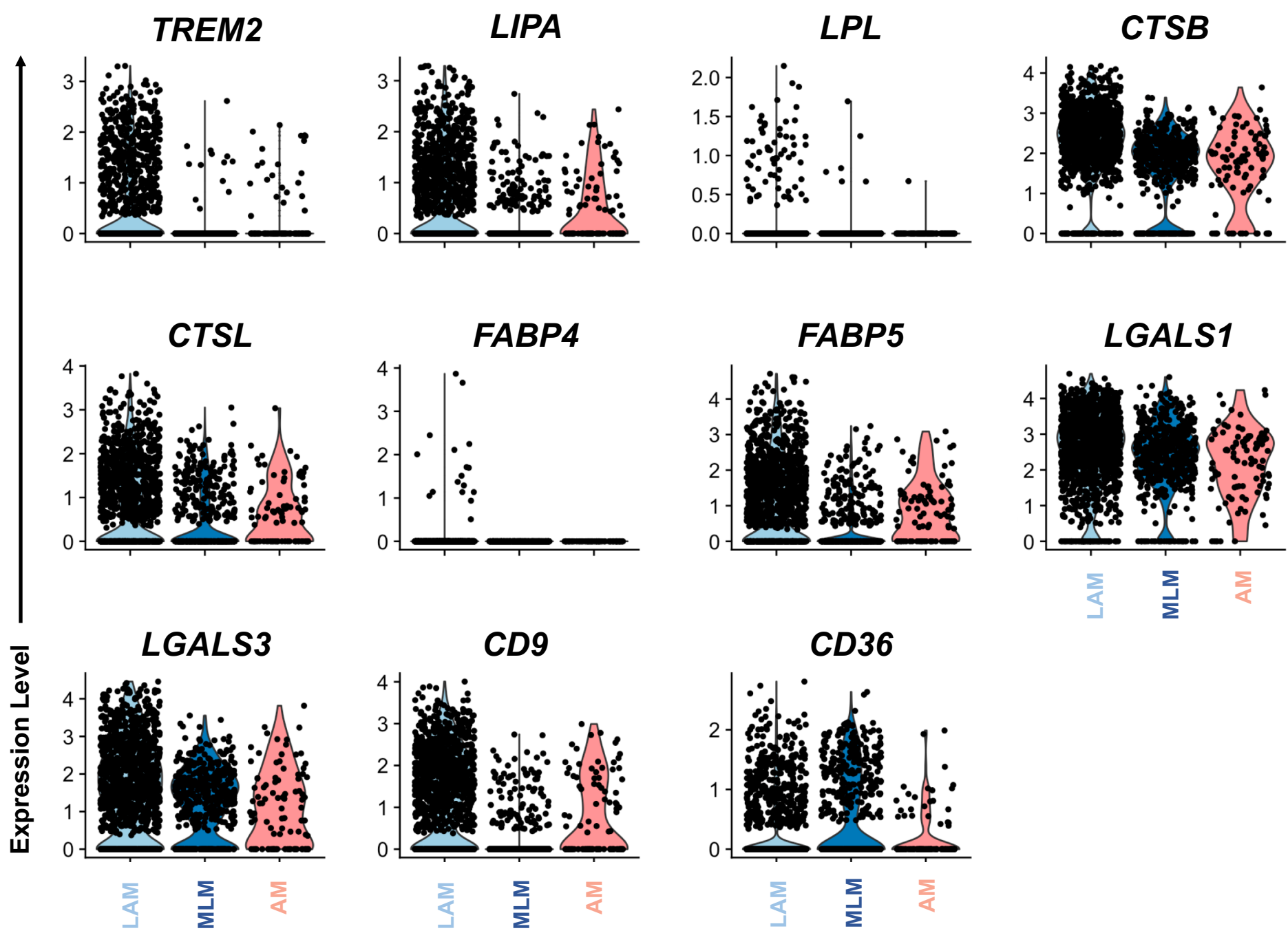

Supplement: S5 Fig — (PDF) [file pone.0244743.s005.pdf]

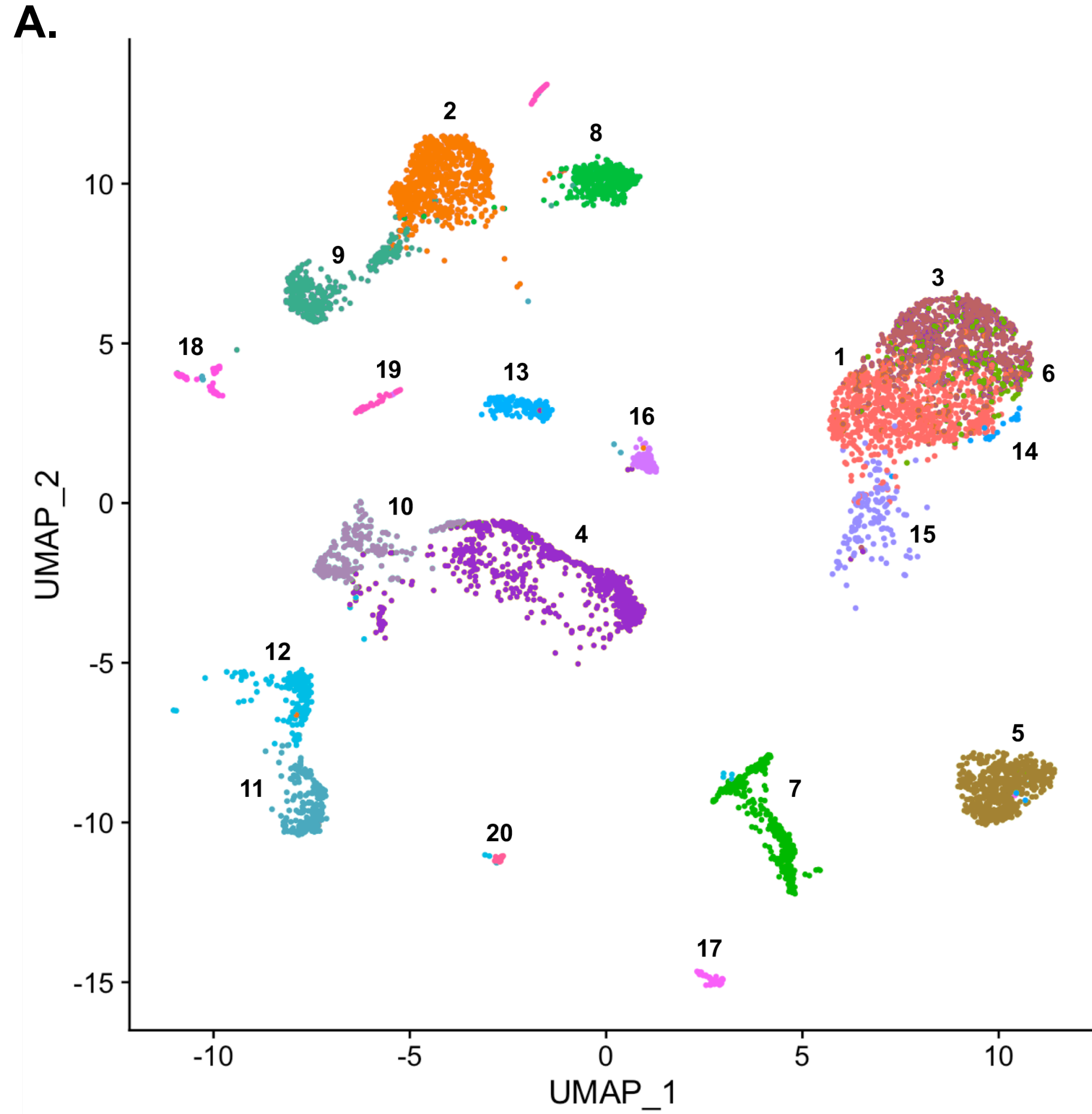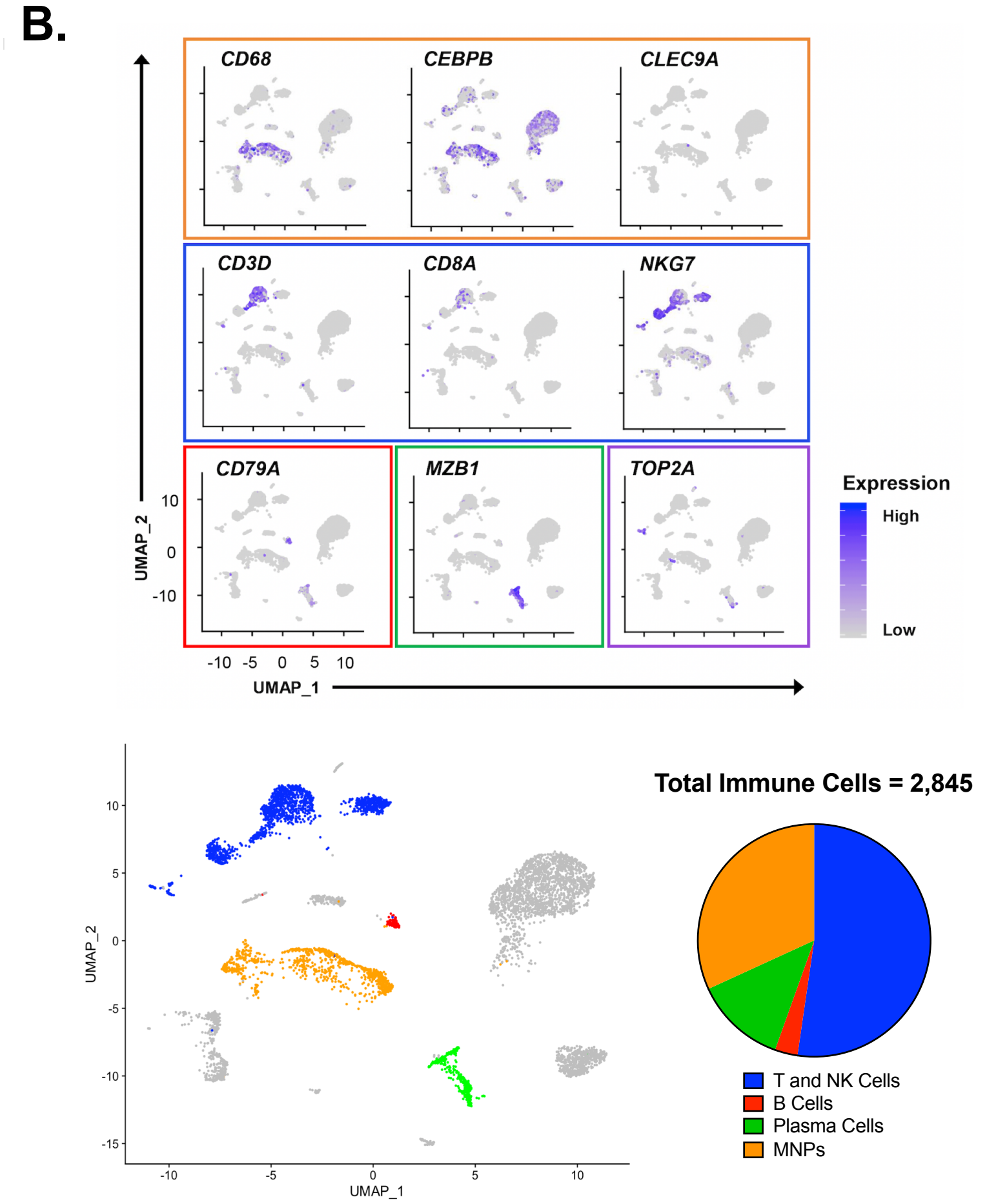

Supplement: S6 Fig — UMAP reproducing the 20 clusters of cells, including inflammatory macrophages (IM, cluster 4) and non-inflammatory macrophages (NM, cluster 10), from previously published scRNA-seq of non-diseased livers [21] (A). Expression of lineage-specific genes verifies the identify of immune cells clusters (blue = T/NK cells; red = B cells; green = plasma cells; purple = dividing cells; orange = MNP). The UMAP is recolored by cell type and the proportion of immune cells is shown (B). (PDF) [file pone.0244743.s006.pdf]

**A.**

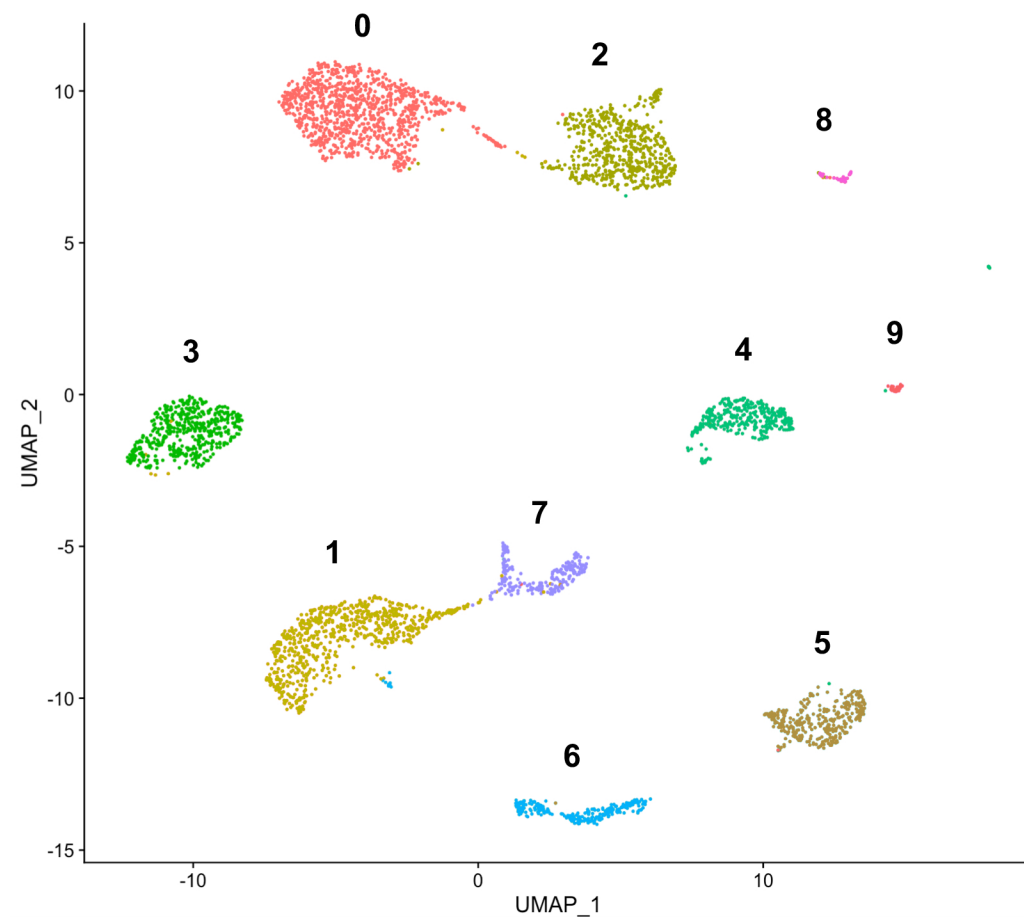

**B.**

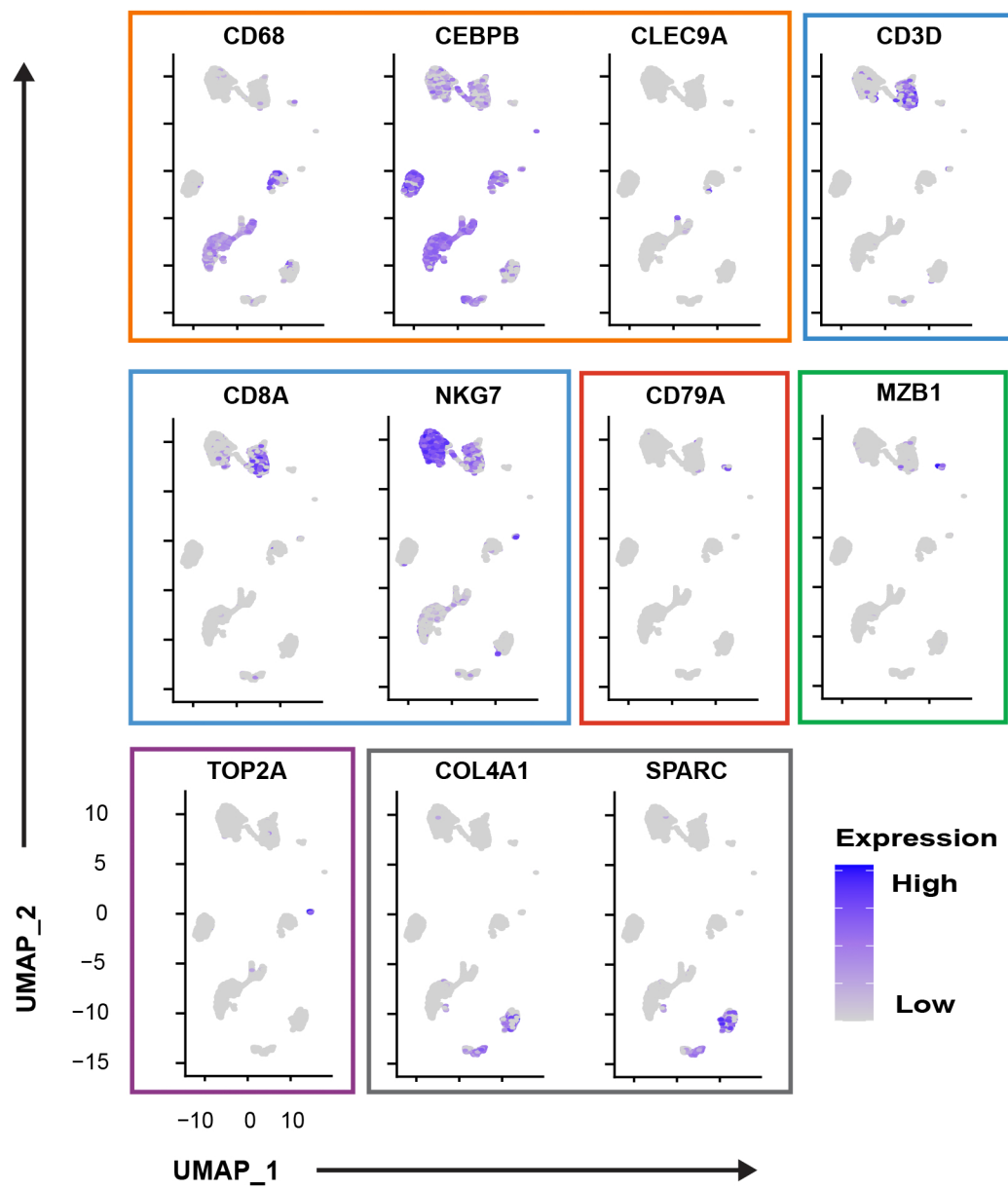

**C.**

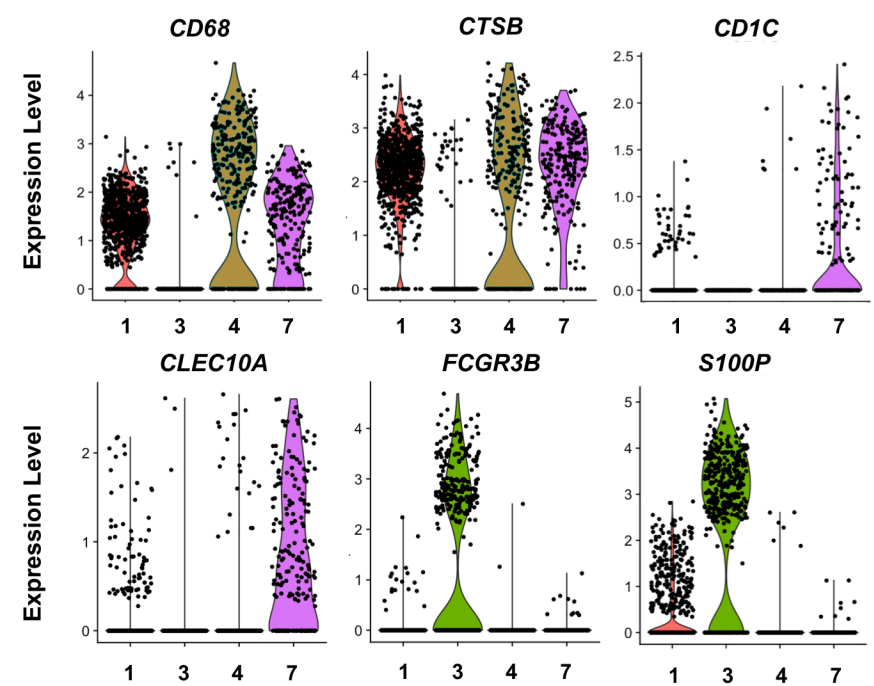

**D.**

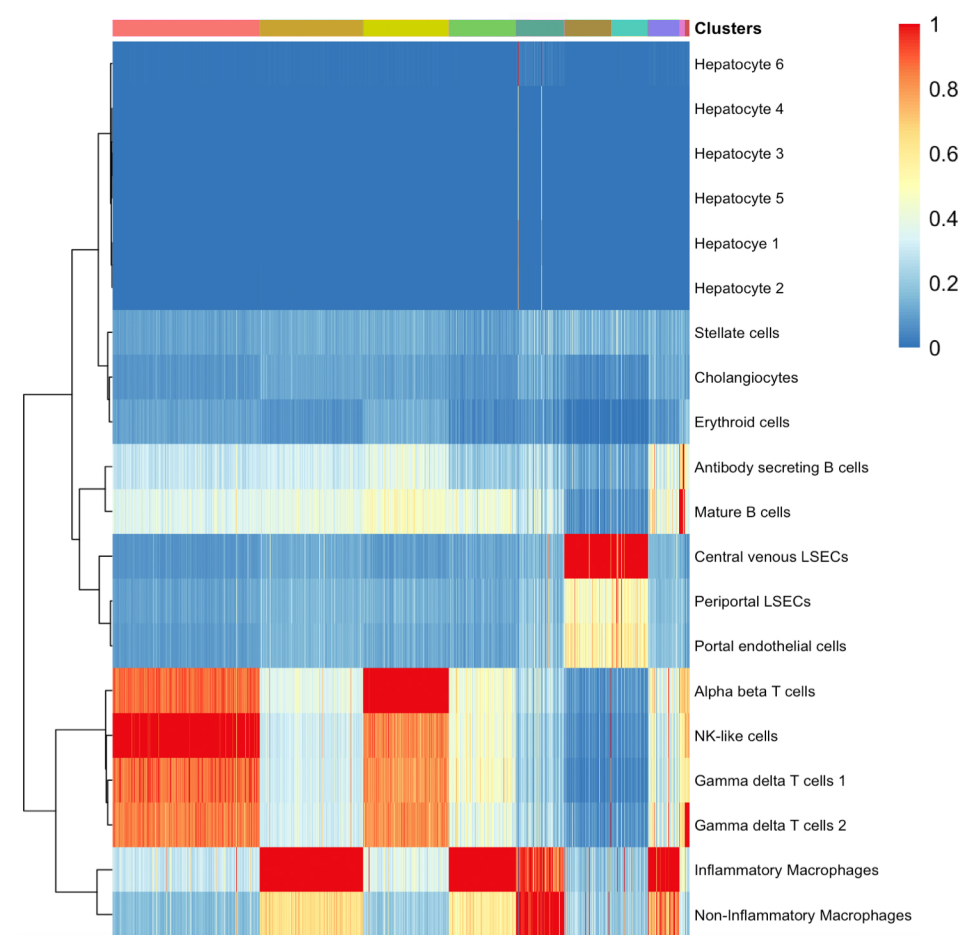

**E.**

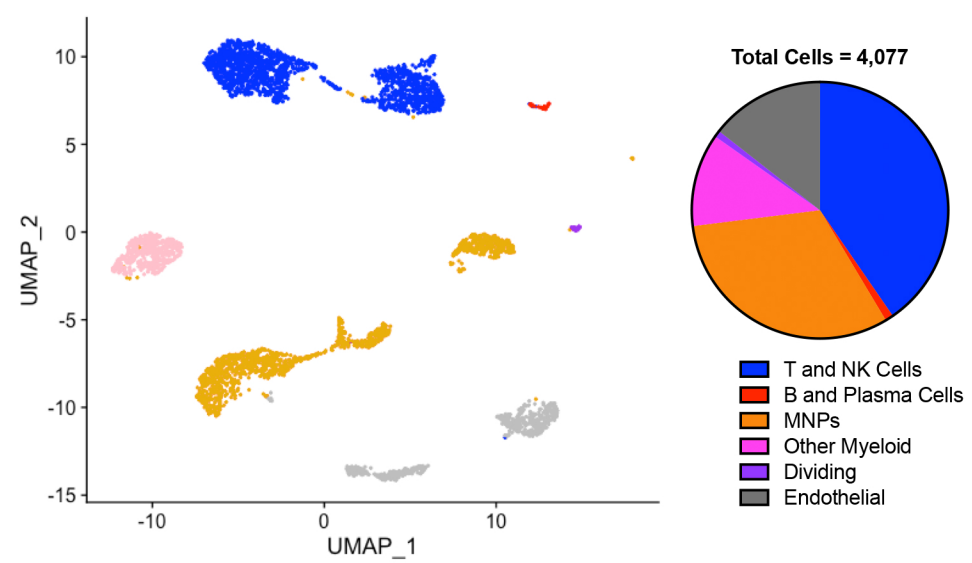

Supplement: S7 Fig — UMAP of scRNA-seq data from a pediatric non-cholestatic liver (NC) shows 10 clusters of cells (A). Feature plot demonstrates expression of lineage-specific genes by cell cluster (blue = T/NK cells; red = B cells; green = plasma cells; orange = MNP; pink-other myeloid cells; purple = dividing cells; gray = endothelial cells) (B). Comparison of gene expression across all myeloid cell clusters identifies cluster 3 as neutrophils expressing FCGR3B and S100P, CD1c+ dendritic cells as cluster 7, and cluster 1 and 4 as macrophage clusters (C). Single-R analysis using previously published data from adult normal livers as the reference [21] supports our cluster assignments with the addition of neutrophil and dividing cell clusters (D). Re-colored UMAP by cell type and proportion of immune cells demonstrates high numbers of MNP and T/NK cells with contribution of endothelial cells from possible contamination (E). (PDF) [file pone.0244743.s007.pdf]
