# Supplementary material for: Transcriptional profiling of pediatric cholestatic livers identifies three distinct macrophage populations
Source: PLoS One. 2021 Jan 7;16(1):e0244743. doi: 10.1371/journal.pone.0244743 (PMC7790256; doi:10.1371/journal.pone.0244743)

A.

	Gender	Age at OLT	TB (mg/dL)	DB (mg/dL)	GGT (IU/L)	AST (IU/L)	ALT (IU/L)	INR	Platelets (10^3 cells/μL)	Estimated Number of Cells	Median UMI Counts/Cell	Median Genes/Cell	Reads Mapped to Genome	Fraction of Reads in Cells
**ALGS**	M	22 mos	17.8	12.9	411	381	375	1.1	301	5,027	950	444	92.8%	87.8%
**BASM**	F	6 mos	15.3 (25.5)	4.2 (16.2)	24	307	118	1.6 (3.9)	155	2,633	1,695	636	96.4%	86.1%
**iBA**	F	10 mos	17.1	11.4	34	269	134	1.3	221	5,927	4,742	1,753	90.9%	91.2%
**NC**	F	133 mos	0.7	<0.2	53	104	74	1.3	278	4,691	4,600	1,617	92.9%	89.3%

B.

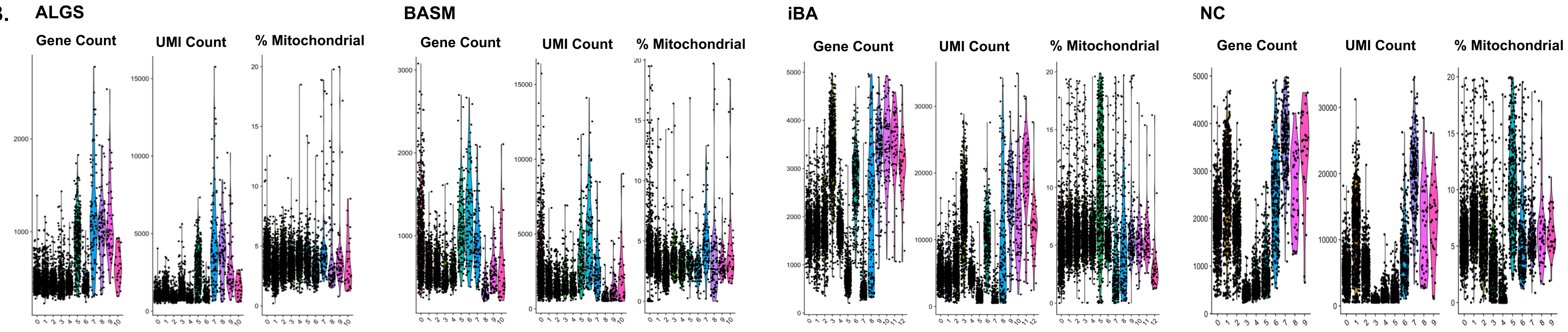

C.

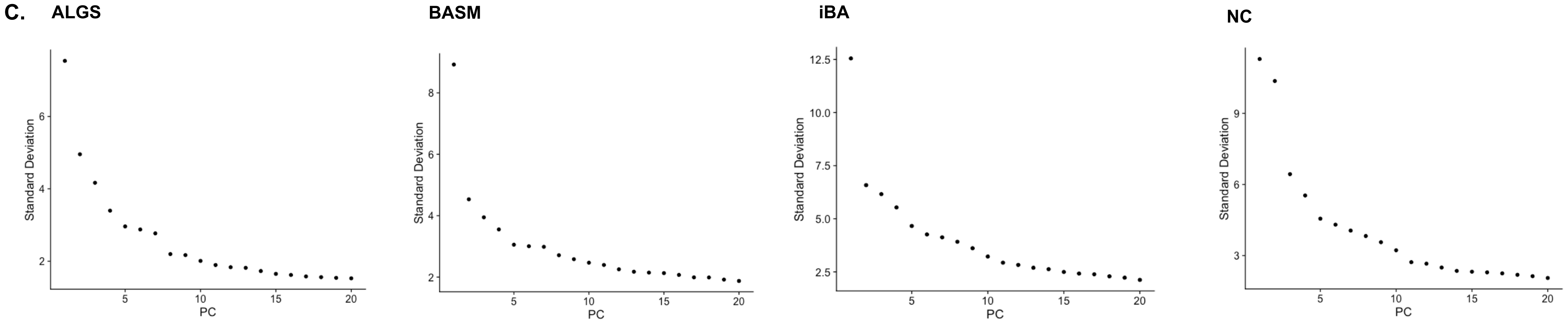

Supplement: S2 Fig — Demographic, laboratory, and sequencing data is provided for each patient (A). Age was rounded down to the nearest month. Reported laboratory values were obtained within 24 hours prior to liver transplant. Admission bilirubin levels and INR obtained 1 week prior to liver transplant before initiation of fresh frozen plasma and renal replacement therapy are reported in parentheses for BASM. Distribution of gene counts, unique molecular identifier (UMI) counts, and percent mitochondrial genes per cell in ALGS, BASM, iBA and NC (left to right) are shown by cluster (B). The standard deviation associated with each principal component (PC) in the analysis of each single-cell RNA-seq dataset (C). (PDF) [file pone.0244743.s002.pdf]
