# Supplementary material for: Transcriptional profiling of pediatric cholestatic livers identifies three distinct macrophage populations
Source: PLoS One. 2021 Jan 7;16(1):e0244743. doi: 10.1371/journal.pone.0244743 (PMC7790256; doi:10.1371/journal.pone.0244743)
Supplement: S9 Fig — Macrophages from non-diseased liver (NL) were categorized into 5 groups based on their inferred pseudotime (A). From 23 modules of genes with pseudotime-associated expression, we chose module 4 with increased expression at pseudotime 0–5 to represent the non-inflammatory module and module 2 with increased expression at pseudotime 20–25 to represent the inflammatory module (B). Visualization of gene expression for these 2 modules in non-cholestatic pediatric liver macrophages shows that module 4 is upregulated in Ped1 macrophages similar to non-inflammatory adult macrophages and module 2 is upregulated in Ped2 macrophages similar to inflammatory adult macrophages (IM) (C). Comparison to cholestatic macrophages demonstrated low expression of the non-inflammatory module across all subsets whereas MLM demonstrated high expression of the inflammatory module (D). (PDF) [file pone.0244743.s009.pdf]

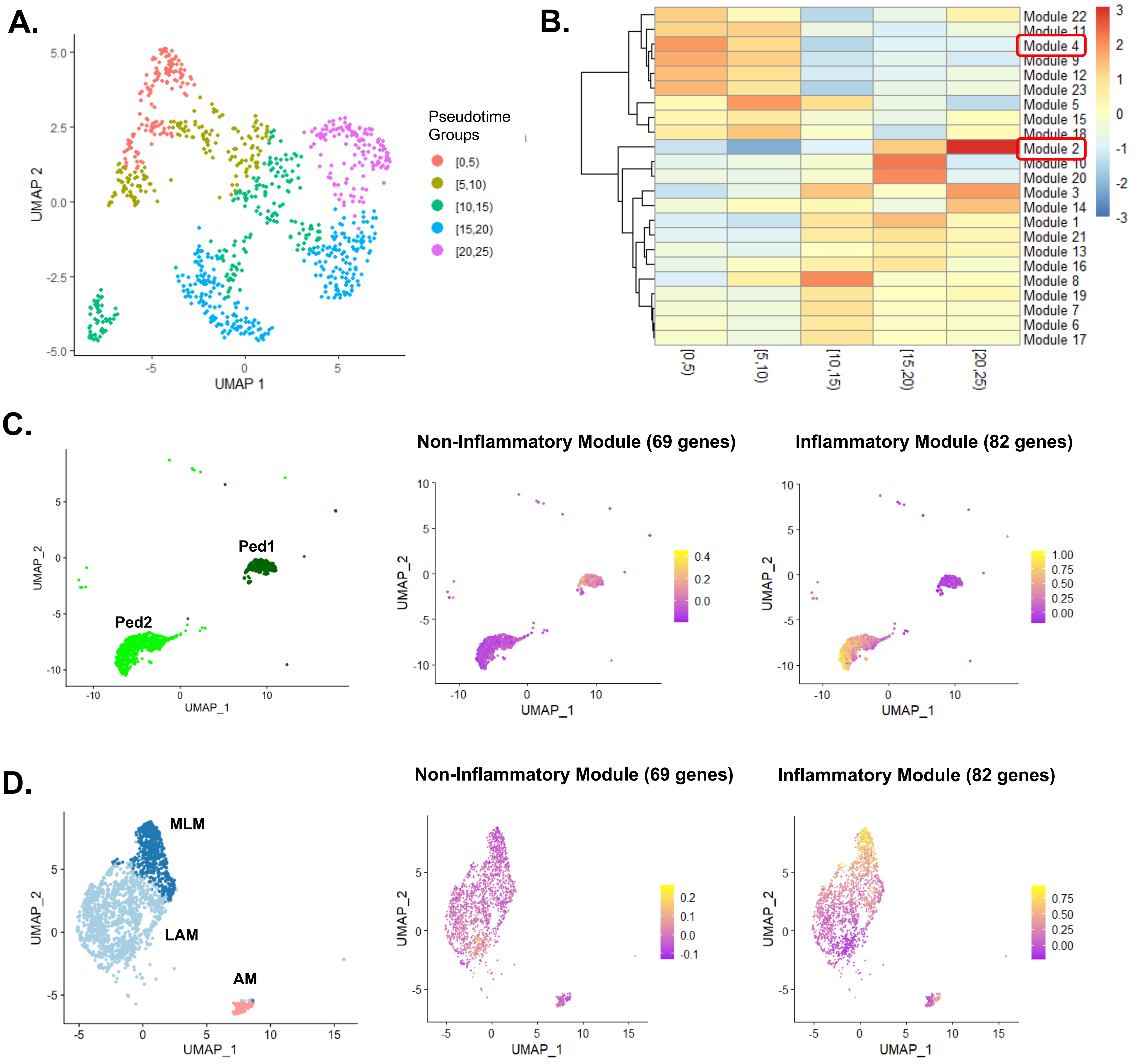

A.
Pseudotime Groups
[0,5)
[5,10)
[10,15)
[15,20)
[20,25)
UMAP 1
UMAP 2
B.
Module 22
Module 11
Module 4
Module 9
Module 12
Module 23
Module 5
Module 15
Module 18
Module 2
Module 10
Module 20
Module 3
Module 14
Module 1
Module 21
Module 13
Module 16
Module 8
Module 19
Module 7
Module 6
Module 17
[0,5)
[5,10)
[10,15)
[15,20)
[20,25)
C.
Ped1
Ped2
Non-Inflammatory Module (69 genes)
Inflammatory Module (82 genes)
UMAP_1
UMAP_2
D.
MLM
LAM
AM
Non-Inflammatory Module (69 genes)
Inflammatory Module (82 genes)
