# Supplementary material for: Transcriptional profiling of pediatric cholestatic livers identifies three distinct macrophage populations
Source: PLoS One. 2021 Jan 7;16(1):e0244743. doi: 10.1371/journal.pone.0244743 (PMC7790256; doi:10.1371/journal.pone.0244743)
Supplement: S8 Fig — NR1H4 encoding FXR was absent in cholestatic macrophages. (PDF) [file pone.0244743.s008.pdf]

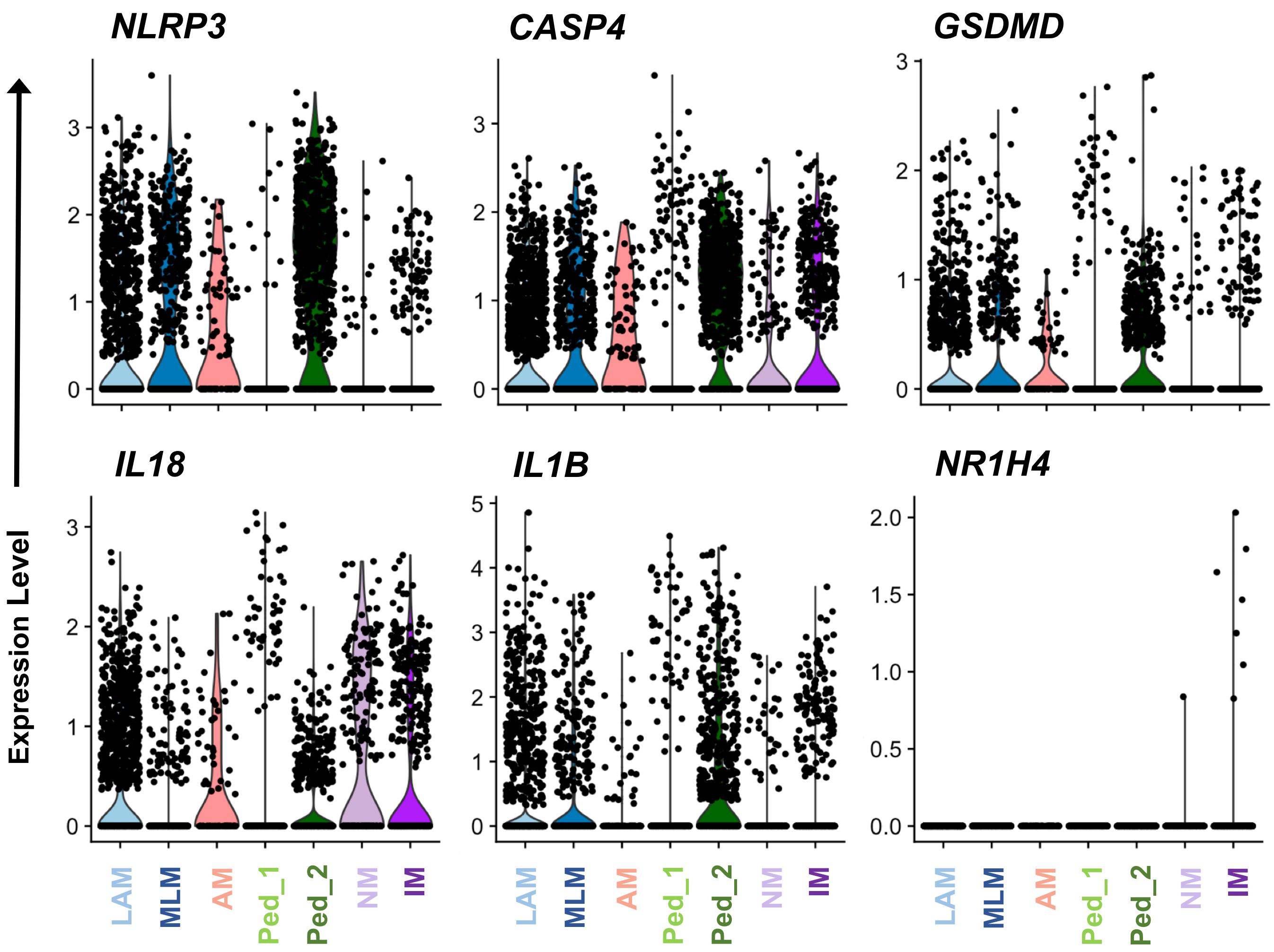
NLRP3
CASP4
GSDMD
IL18
IL1B
NR1H4
Expression Level
LAM
MLM
AM
Ped_1
Ped_2
NM
IM
